# Supplementary material for: Genomic analyses of a livestock pest, the New World screwworm, find potential targets for genetic control programs
Source: Commun Biol. 2020 Aug 4;3:424. doi: 10.1038/s42003-020-01152-4 (PMC7403345; doi:10.1038/s42003-020-01152-4)
Supplement: Supplementary file 1 — Supplementary Information [file 42003_2020_1152_MOESM1_ESM.pdf]

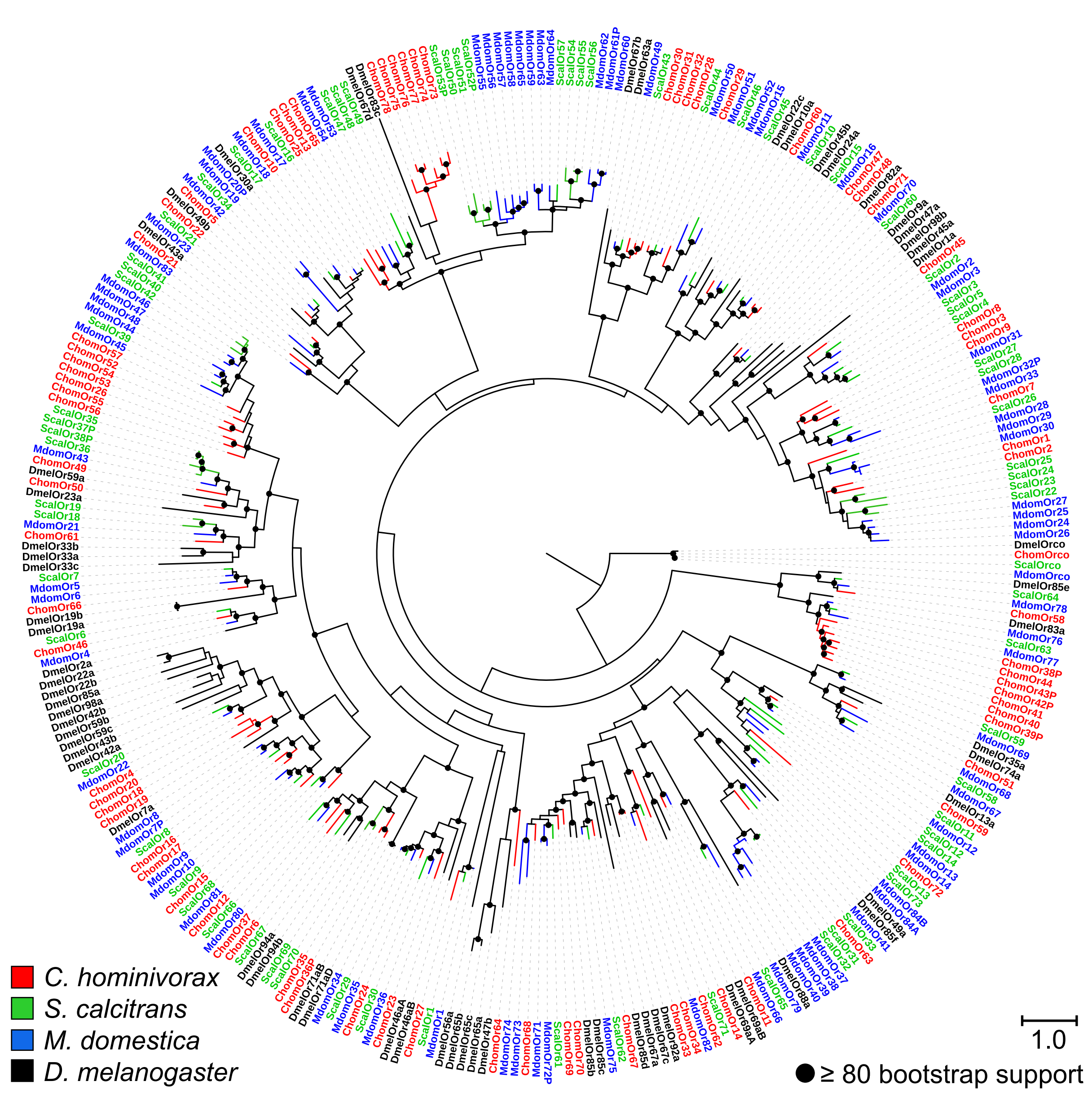

Supplementary Fig. 1. Phylogenetic relationships among odorant receptors (ORs) in the screw worm fly (*C. hominivorax*), house fly (*M. domestica*), stable fly (*S. calcitrans*) and fruit fly (*D. melanogaster*). Phylogeny estimated using the Maximum Likelihood method in RAxML. The tree is rooted at the Orco clade. Branch support estimated using 500 bootstrap replications.

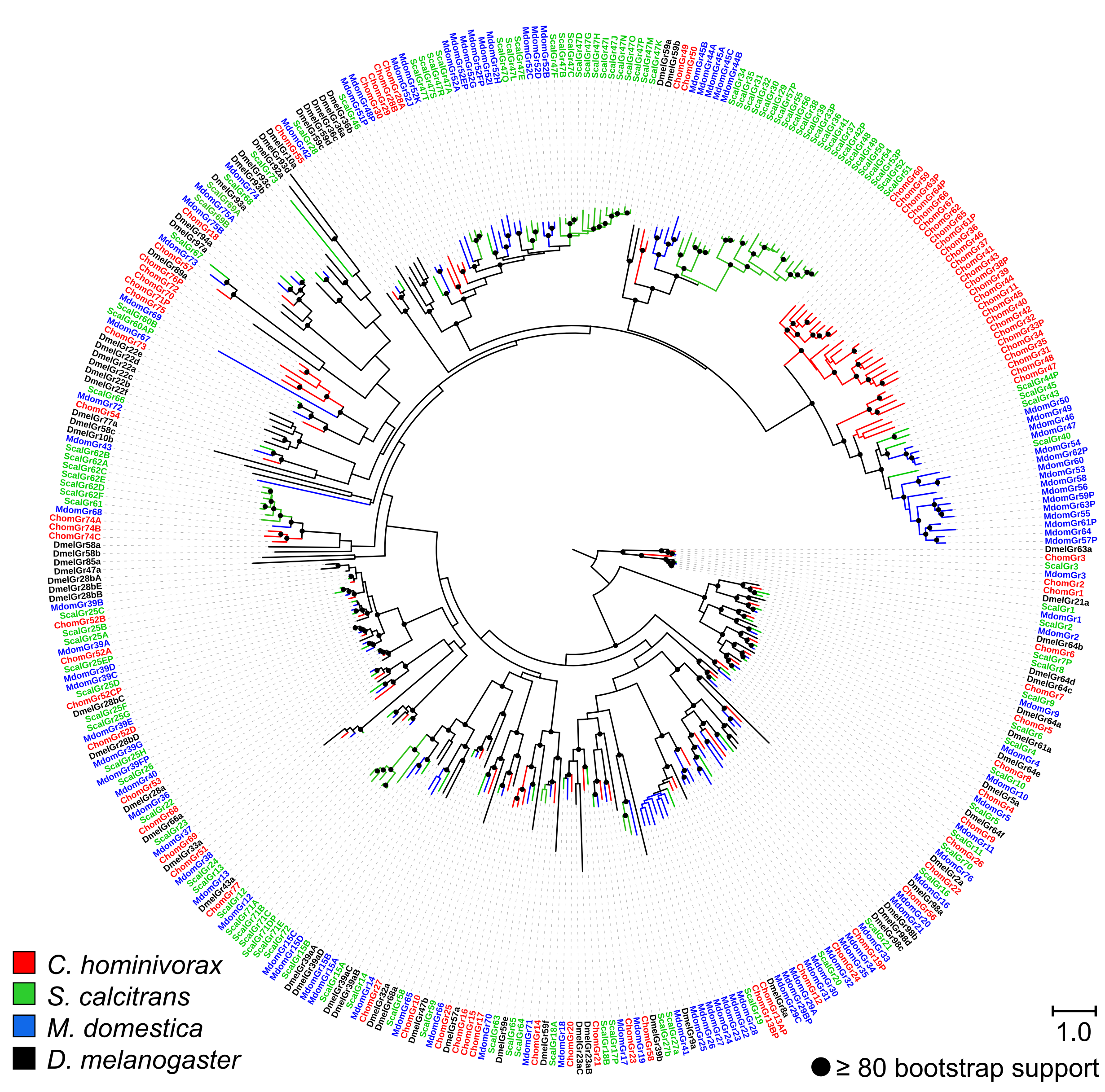

Supplementary Fig 2. Phylogenetic relationships among gustatory receptors (GRs) in the screw worm fly (*C. hominivorax)*, house fly (*M. domestica*), stable fly (*S. calcitrans*) and fruit fly (*D. melanogaster*). Phylogeny estimated using the Maximum Likelihood method in RAxML. The tree is rooted at the CO2 receptor clade. Branch support estimated using 500 bootstrap replications.

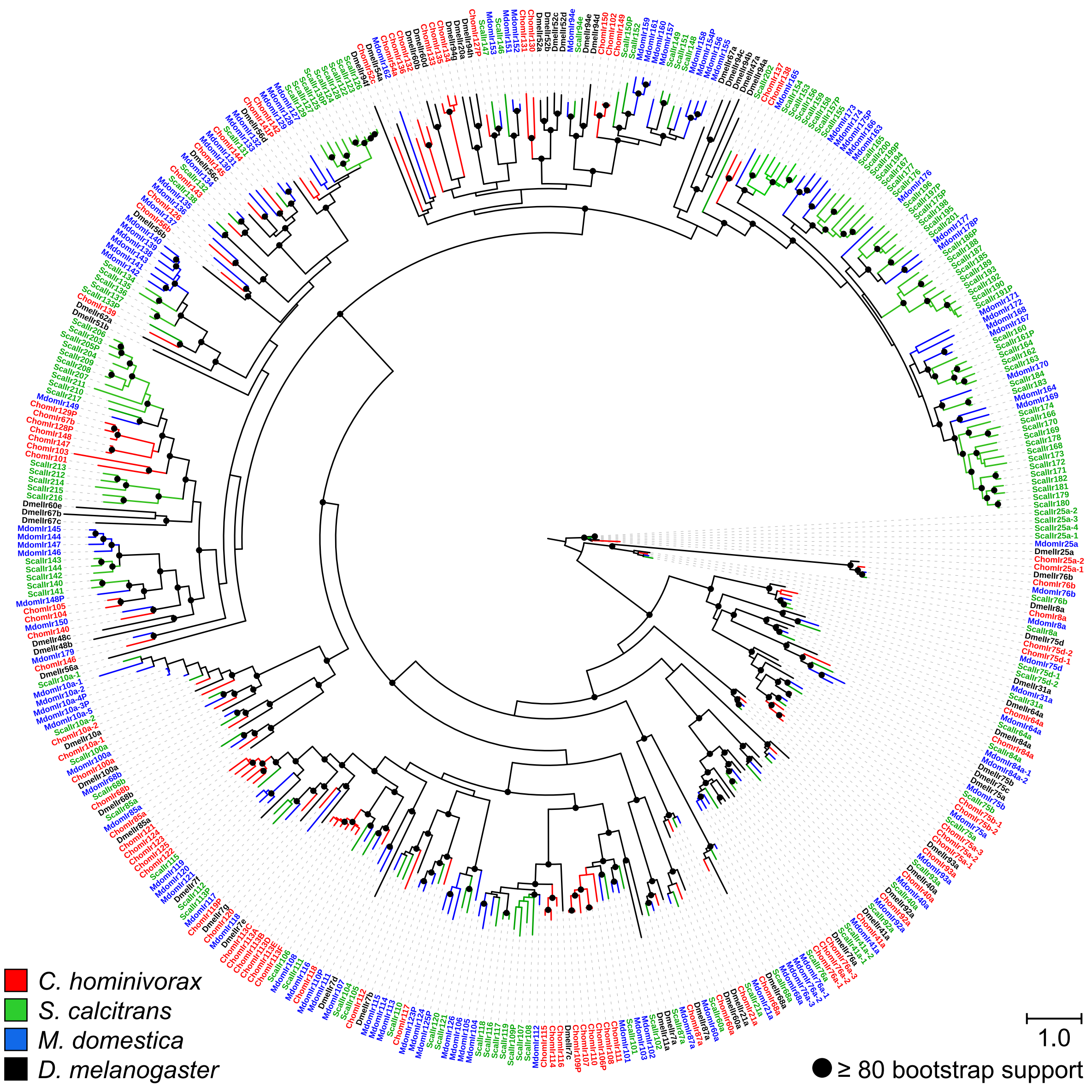

Supplementary Fig. 3. Phylogenetic relationships among ionotropic receptors (IRs) in the screw worm fly (*C. hominivorax*), house fly (*M. domestica*), stable fly (*S. calcitrans*) and fruit fly (*D. melanogaster*). Phylogeny estimated using the Maximum Likelihood method in RAxML. The tree is rooted at the Ir25a clade. Branch support estimated using 500 bootstrap replications.

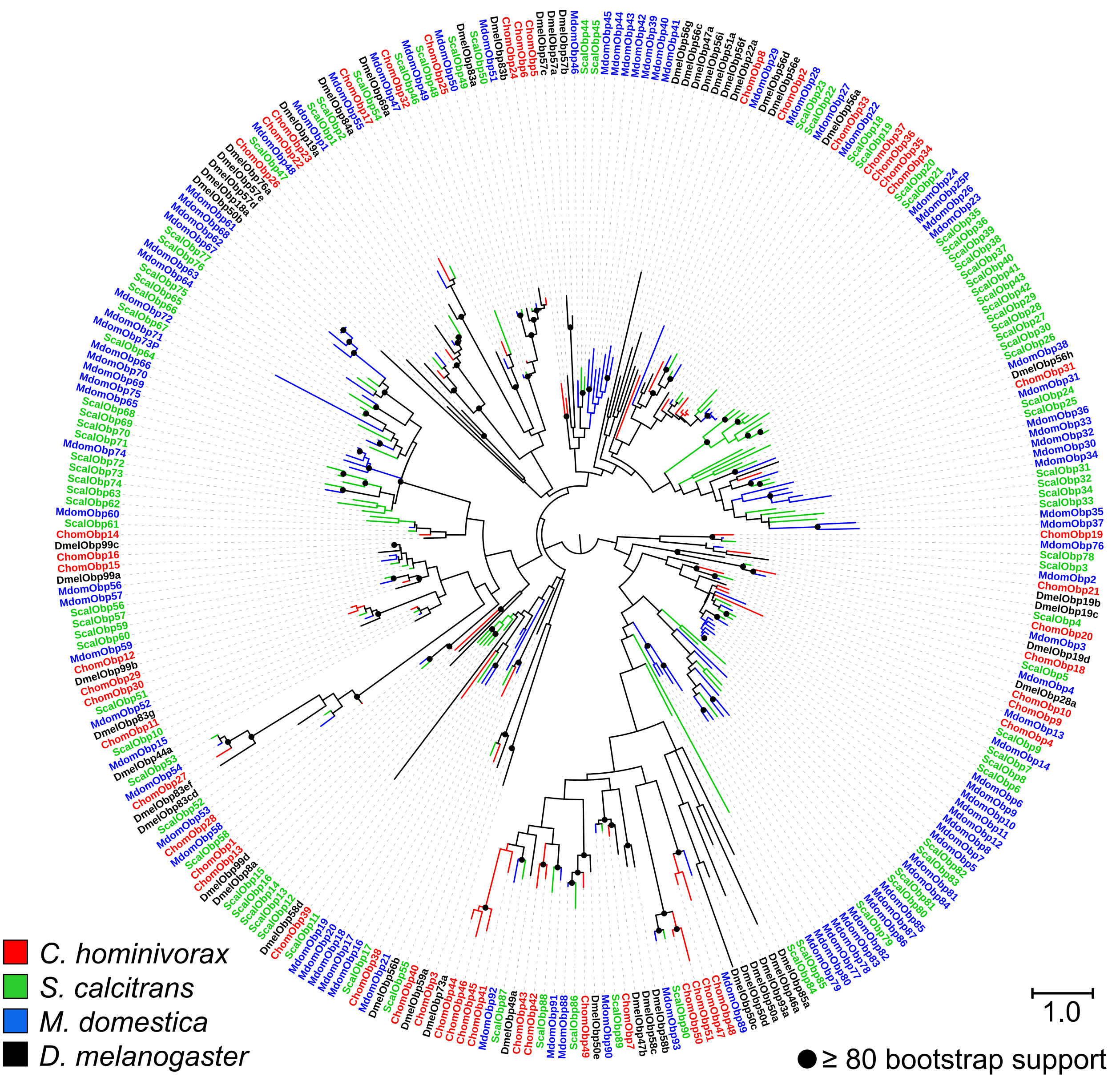

Supplementary Fig. 4. Phylogenetic relationships among odorant binding proteins (OBPs) in the screw worm fly (*C. hominivorax*), house fly (*M. domestica*), stable fly (*S. calcitrans*) and fruit fly (*D. melanogaster*). Phylogeny estimated using the Maximum Likelihood method in RAxML. The tree is rooted at the midpoint. Branch support estimated using 500 bootstrap replications.

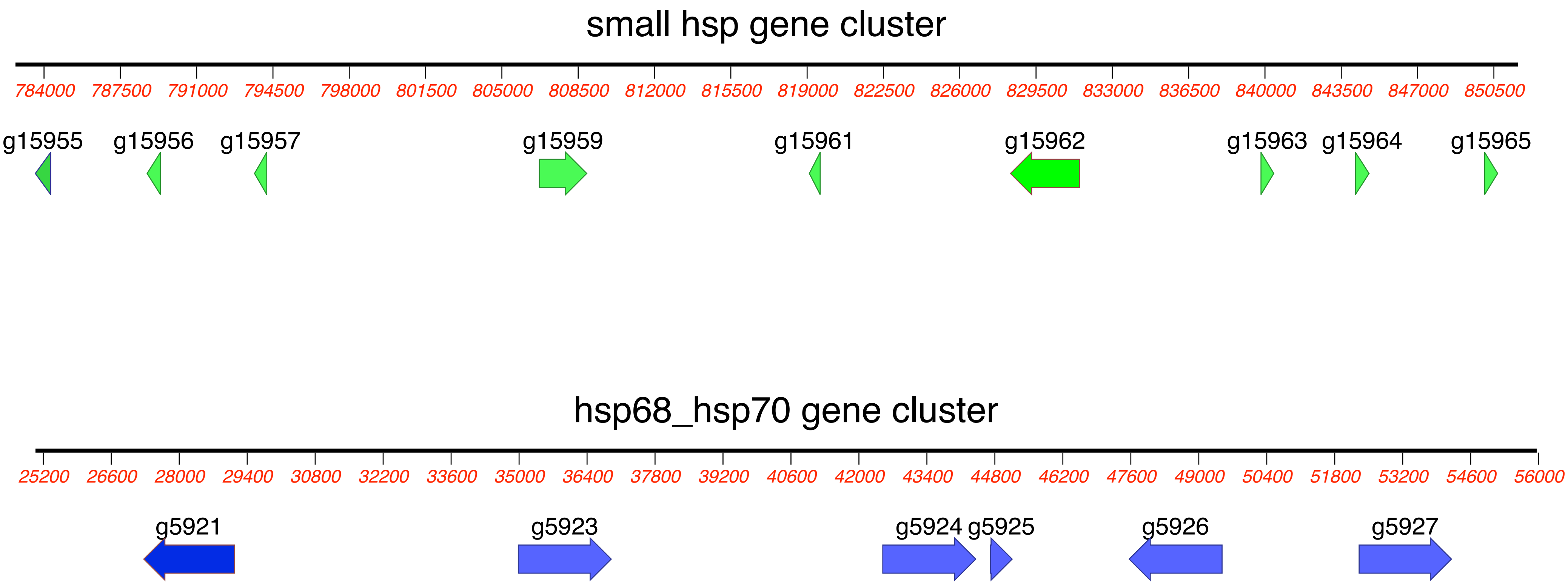

Supplementary Fig 5. Heat shock protein gene clusters. Schematic illustration of small hsp and hsp68/70 gene clusters. Genes are shown as arrows pointing in a 5'-3' direction.

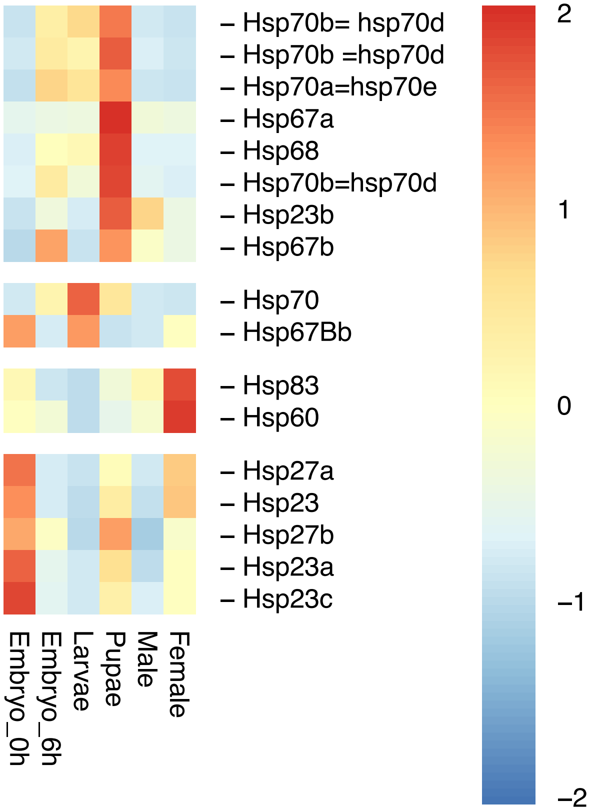

Supplementary Fig 6. Heat map of heat shock protein genes developmental expression.

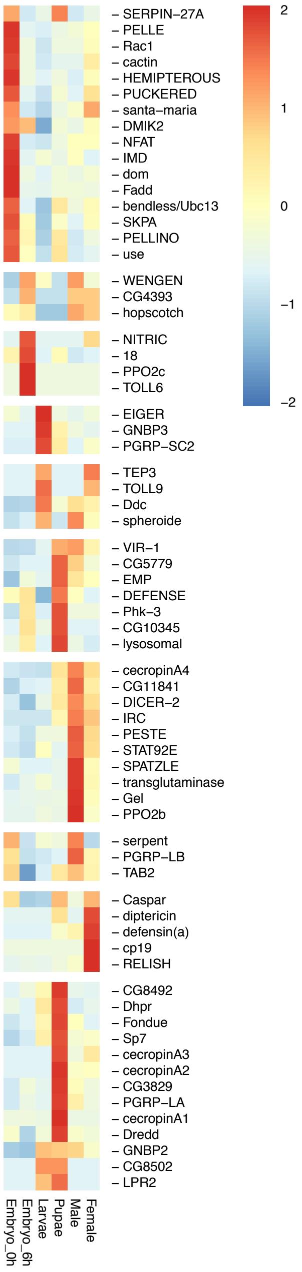

Supplementary Fig 7. Heat map of immune response genes developmental expression.

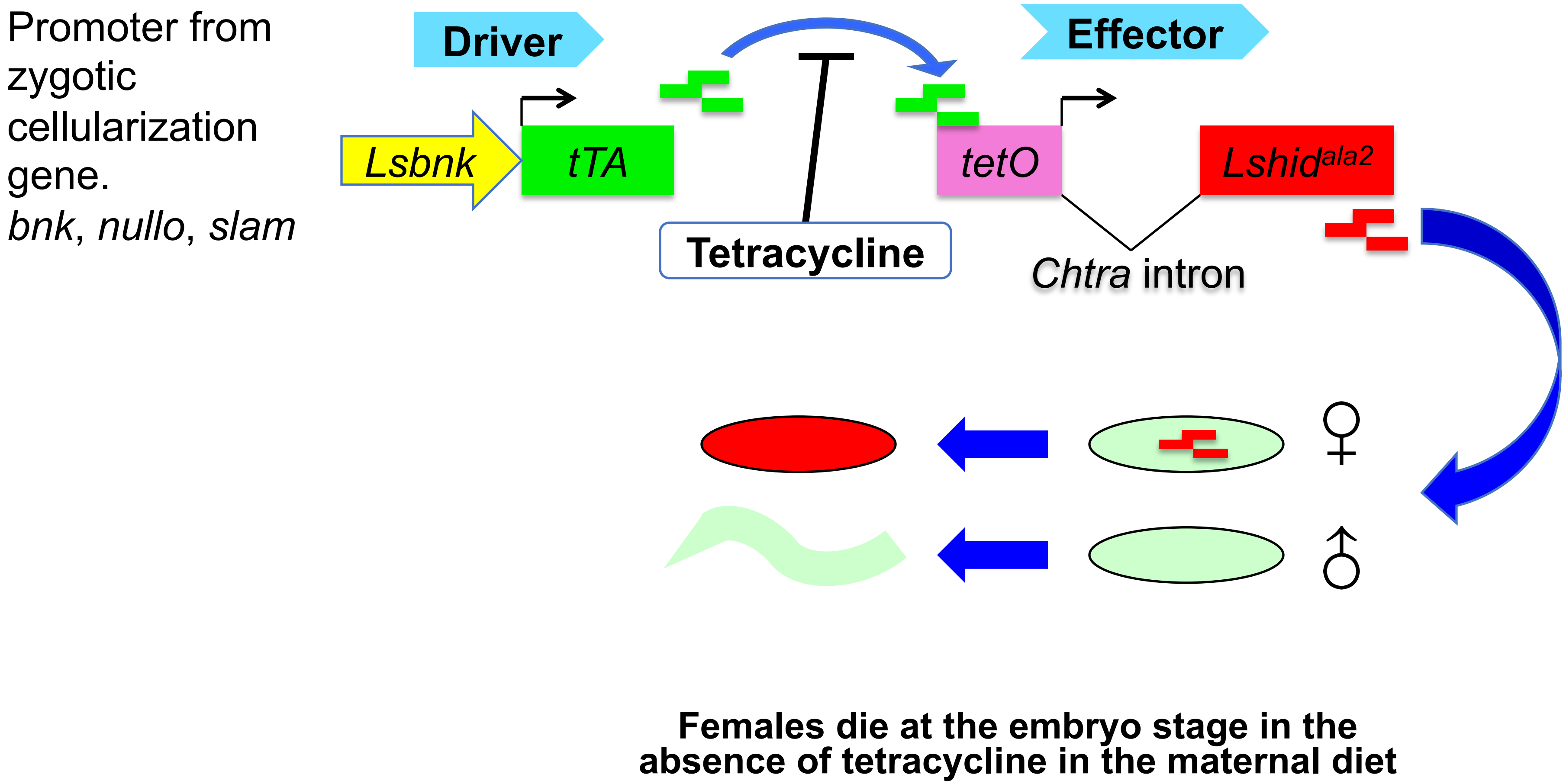

Supplementary Fig 8. Tetracycline-repressible transgenic embryonic sexing system.

Multi-copy Cellularization Genes

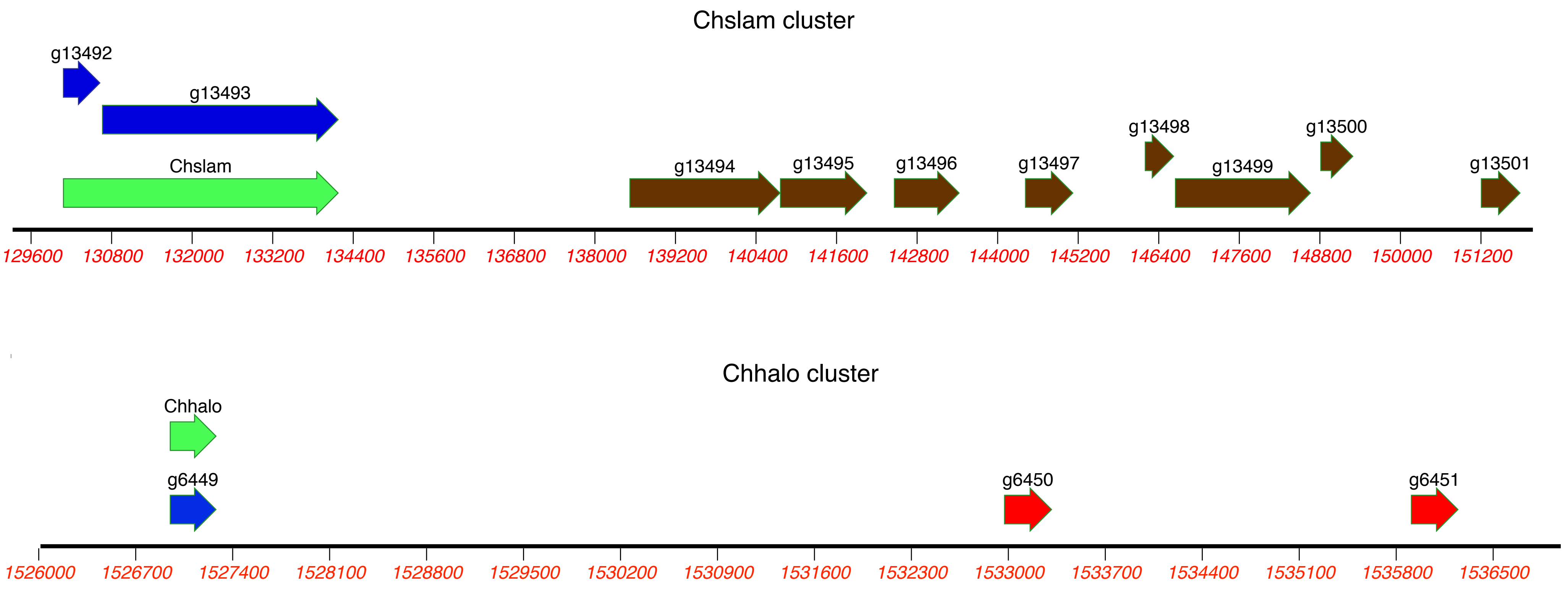

Supplementary Fig. 9. *Chslam* and *Chhalo* gene clusters. Schematic illustration of the *Chslam* and *Chhalo* gene clusters. Genes are shown as arrows pointing in a 5'-3' direction.

hypothetical protein FF38_12096 [*Lucilia cuprina*] orthologous genes

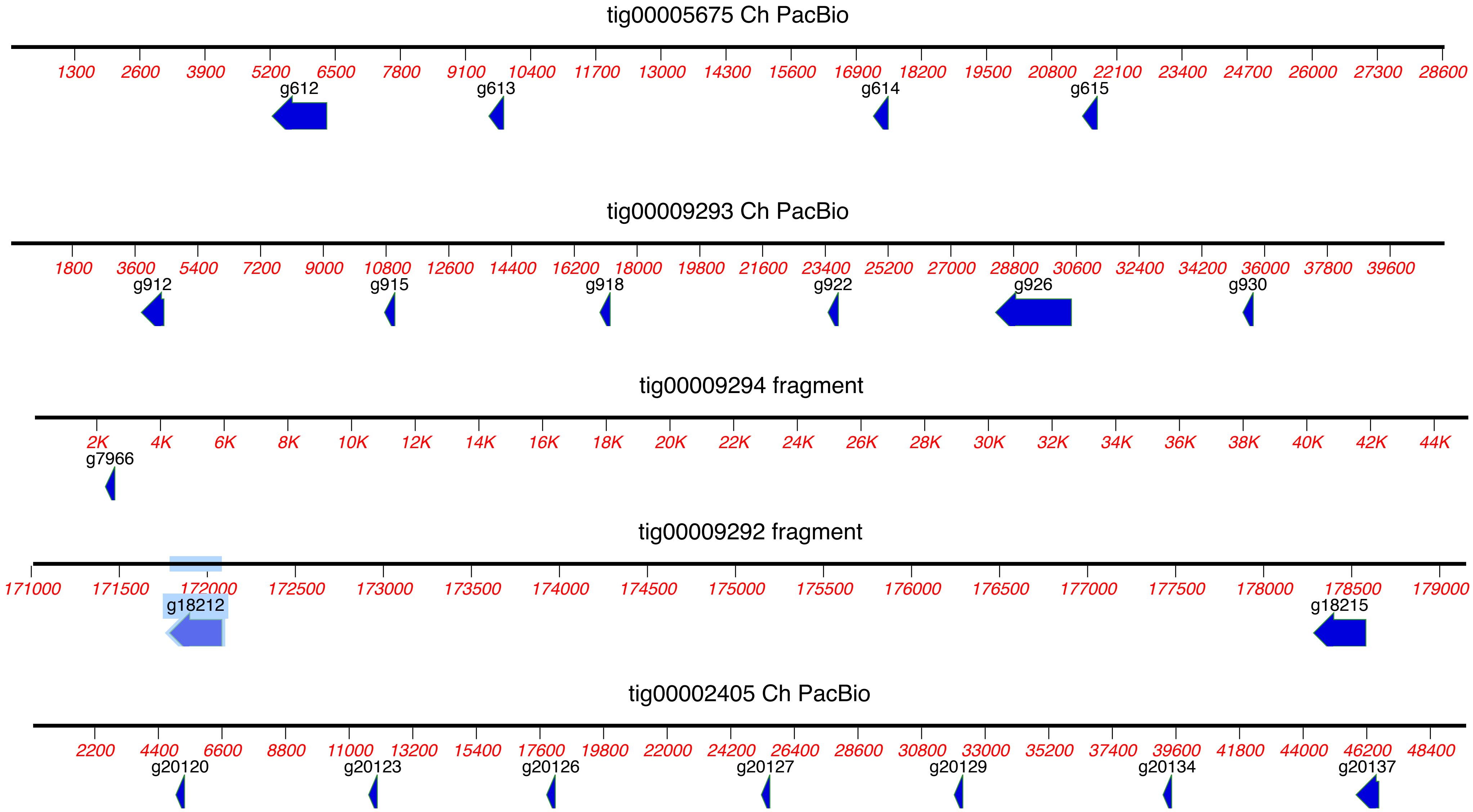

Supplementary Fig. 10. Clusters of genes that encode proteins related to the *L. cuprina* FF38_12096 protein. Schematic illustration of the gene clusters. Genes are shown as arrows pointing in a 5’-3’ direction.

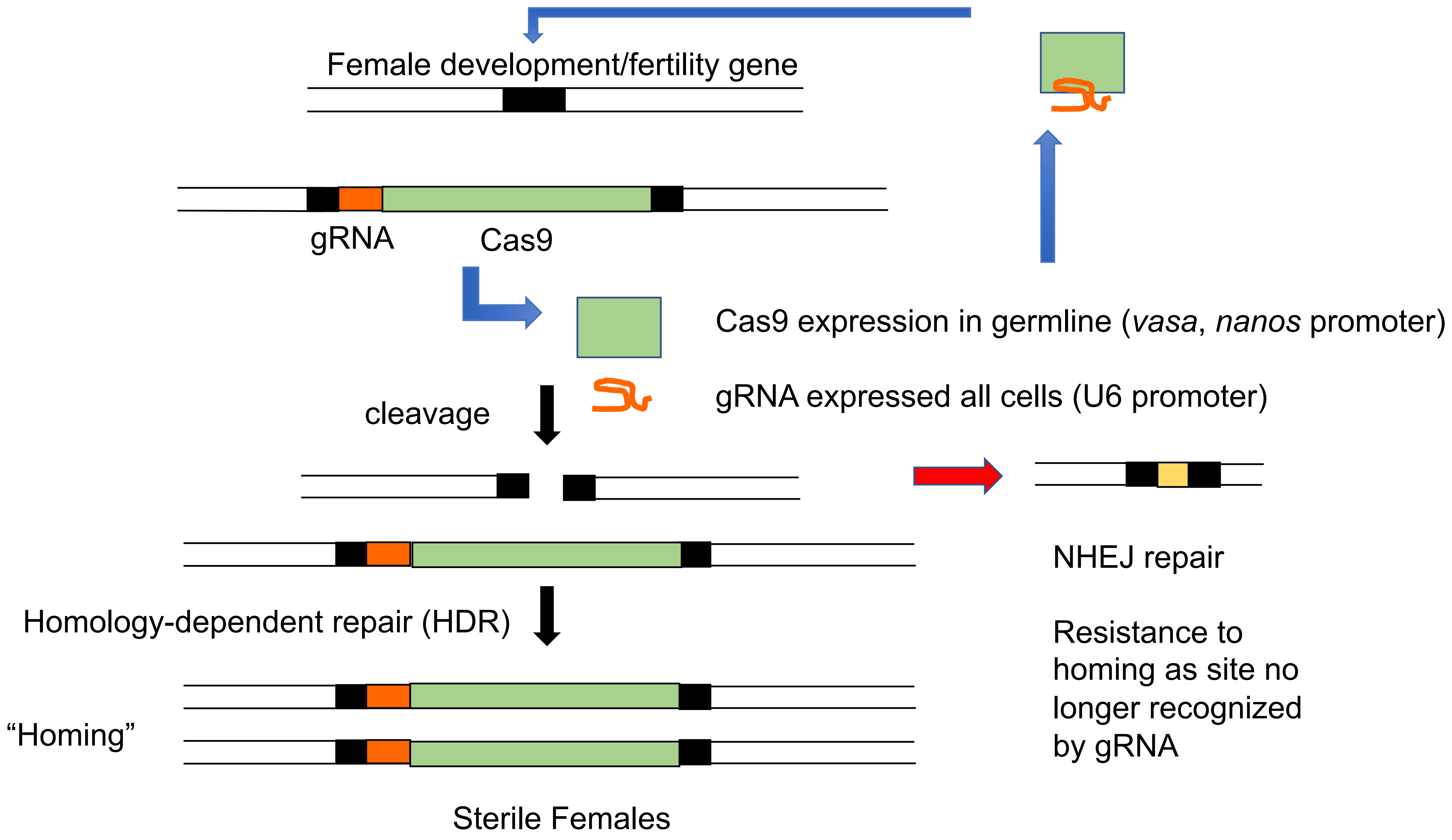

Supplementary Fig. 11. Cas9-mediated homing gene drive for population suppression.

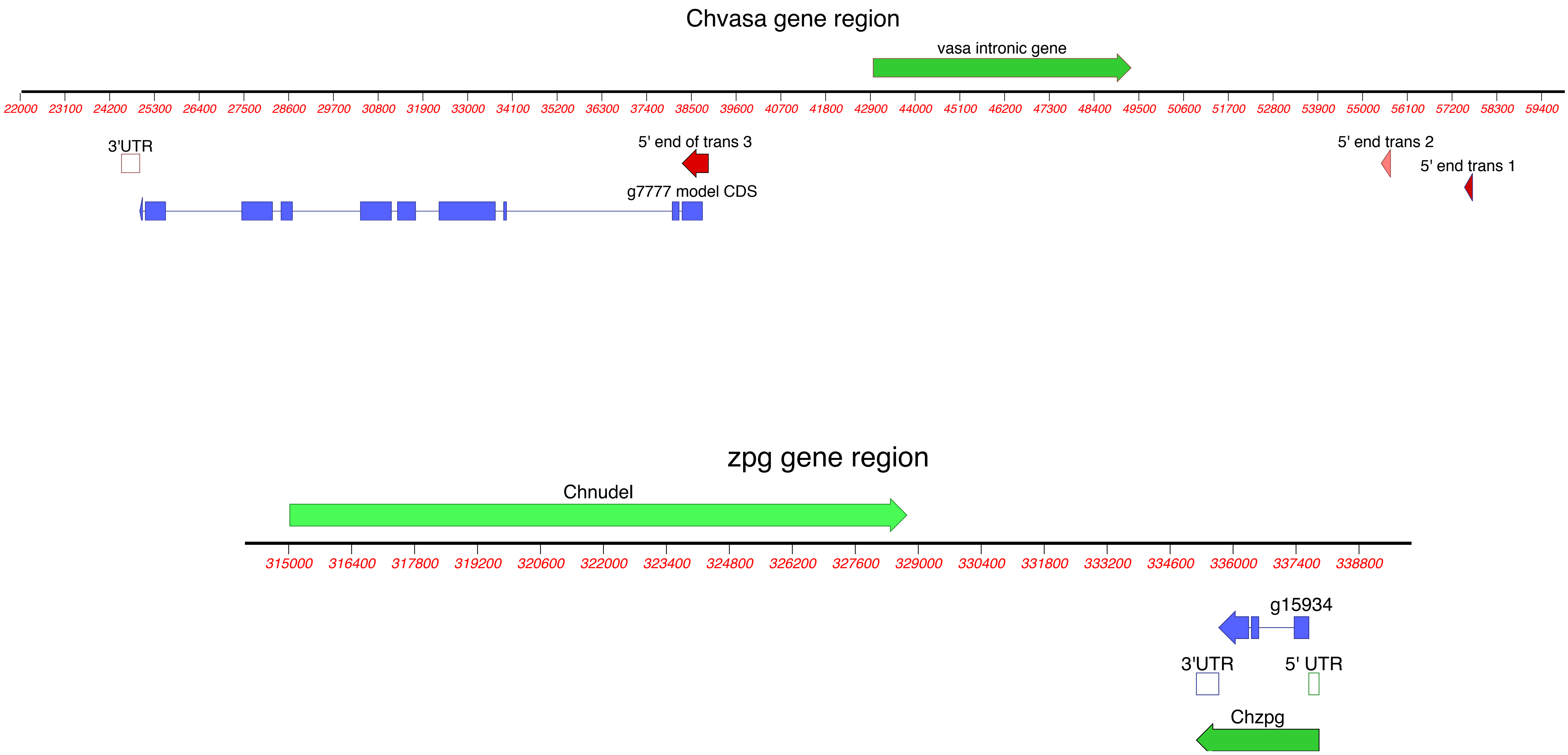

Supplementary Fig. 12. ***Chvasa* and *Chzpg* gene regions.** Schematic illustration of the gene regions with genes shown as arrows pointing in a 5’-3’ direction.

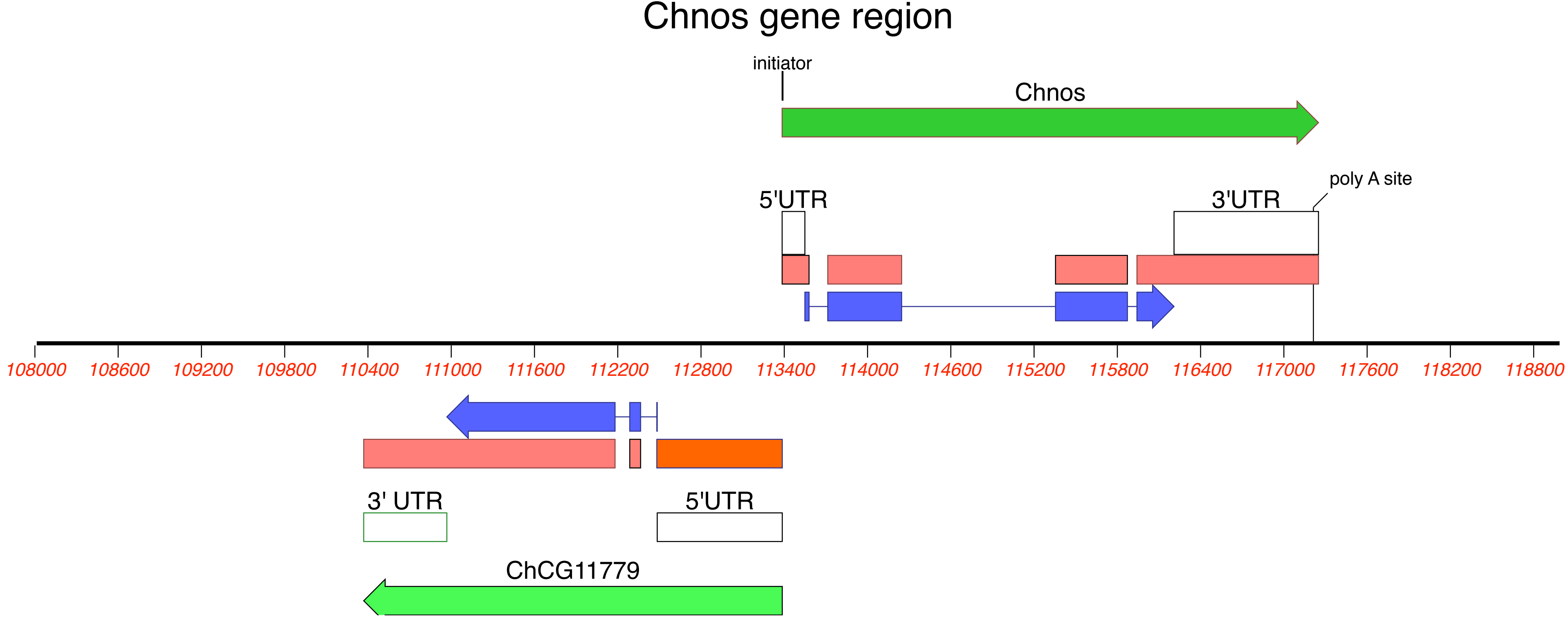

Supplementary Fig. 13. *Chnos* gene region. Schematic illustration of the region with genes shown as arrows pointing in a 5'-3' direction.

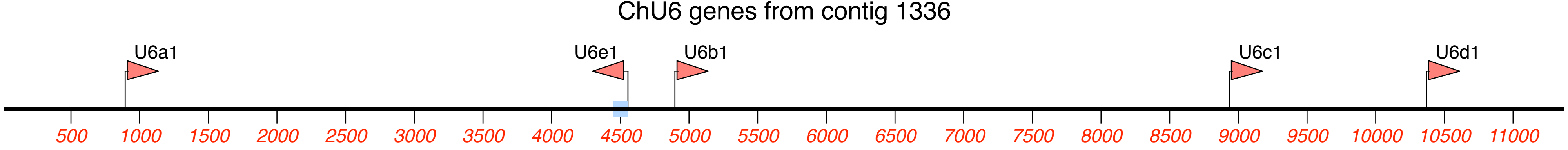

Supplementary Fig 14. U6 snRNA gene region. Schematic illustration of the U6 gene cluster with genes shown as arrows pointing in a 5'-3' direction.
