# Supplementary material for: Genomic analyses of a livestock pest, the New World screwworm, find potential targets for genetic control programs
Source: Commun Biol. 2020 Aug 4;3:424. doi: 10.1038/s42003-020-01152-4 (PMC7403345; doi:10.1038/s42003-020-01152-4)
Supplement: Supplementary file 2 — Description of Additional Supplementary Files [file 42003_2020_1152_MOESM2_ESM.pdf]

Descriptions of Supplementary Data

Supplementary Data 1. C. hominivorax predicted genes.

Supplementary Data 2. C. hominivorax predicted proteins with BLAST match.

Supplementary Data 3. Summary of the repetitive DNA content of the C. hominivorax genome

Supplementary Data 4. Chemosensory genes. Gene sequences for odorant receptors (ORs), gustatory receptors (GRs), ionotropic receptors (IRs) and odorant binding proteins (OBPs) in the C. hominivorax genome assembly.

Supplementary Data 5. Genes expressed mostly at one stage of development.

Supplementary Data 6. Developmental gene expression of transcription factor and heat shock protein genes.

Supplementary Data 7. Transcription factors with or without predicted motifs.

Supplementary Data 8. Modules of genes with similar expression for each developmental stage and sex

Supplementary Data 9. C. hominivorax genes with evidence of positive selection.

Supplementary Data 10. Immune Response Genes.

Supplementary Data 11. Genes that positively regulate apoptosis
