# Supplementary material for: Genomic analyses of a livestock pest, the New World screwworm, find potential targets for genetic control programs
Source: Commun Biol. 2020 Aug 4;3:424. doi: 10.1038/s42003-020-01152-4 (PMC7403345; doi:10.1038/s42003-020-01152-4)
Supplement: Supplementary file 13 — Supplementary Data 11 [file 42003_2020_1152_MOESM13_ESM.rtf]

Supplementary Table 16. Genes that positively regulate apoptosis. Genes that match Drosophila  proteins with GO term 0043065 “positive regulation of apoptosis” are listed.>g8620.t1 MATCH TO: hid head involution defective [Drosophila melanogaster]MAVPFYLPEGGADDTVSSGGTTSSDGASSSAASSLQSSPNTTSSSTQTPMQSPLQPSEIIMALFNEAFYRYSLNSGSHIFQYPTPPSPSCSVHSPSYTGGGEFIFPRSRSTSRIPRTSVSFAAGDEVNFFHQSAPTSSSSTGGNAQTQTTQAPQSAPPIPNSHHHNGQHPLHGHYPYFGYPHIAVGGNSGGGHYQYTPPPTPNTAAATTSTGTSTTTGAHRSLHRSLSDSARRSRLTSTGEDEREYQSEHETSWDDEFDDRYDNFTAGRERLQEFNGRIPPRKKKKDVPKTKPEKKEQSFTWPAVVTVFVFAMGCGFLVAR>g8140.t1 MATCH TO: rpr reaper [Drosophila melanogaster]MAVAFYIPDQAAMLRKAQQQEQQILAMREAHWRYVASVVYDALKQYLEVTPSILQRSKNNKKLKVTSSRAKYHYRHHHY>g8615.t1 MATCH TO: grim grim [Drosophila melanogaster]MAIAYFIPDQAKLLAGQRSNRGNSNATARSSNNNNNQTSSRLPPSNQGNESAPVNSTVNRGYGTTINITVPTLNTTPQEPLGCWDFLTQVFCHALRFYHESSQLQPTVIQISVNFNSQTTATNSTTTPDTTATTTRNQQQTTPTAETTRDTSSEEQRQTKFSKRKN>g8137.t1 MATCH TO: skl sickle [Drosophila melanogaster]MAIPFYENEEQLQQQQPQQNLEEVDQVDGPIAHPSVYQSYVNDADYLPHHDNAQSVTEEDLMAWKILAIAMCKALKDHYQQHLLTTTTTPATTSSNSSPTENSSTAVIQILPNGQIKPQWNOther positive regulators>g9874.t1 MATCH TO: Atg1 Autophagy-related 1 [Drosophila melanogaster]MLKVEVNDKYCNGGDLADYLSVKGTLSEDTVRLFLIQLAGAMKALYTKGIVHRDLKPQNILLSHNYGKTLPAPSKITLKIADFGFARFLNEGVMAATLCGSPMYMAPEVIMSLQYDAKADLWSLGTIVYQCLTGKAPFYAQTPNELKFYYEQNANLAPKIPNGVSPELRDLLLCLLRRNAKDRISFESFFTHRFLQGKKAATSPGDLPASLAMGTTPPTKSKSPLQQQLEQQQQQQQQHLQQQLQQQKQLQQQQQQQLQKQGADDDFQDENAVSVVENPAICATITNVGILCESENNTESSGSHEDSDDFVLVPKNLPEDQNVIDYEKKHSAKQQQQQQNANTAAQNQSSPPRPSTLPISEPKPVPAPVRRYSTNSTANAVAATAPNASPLDKNQVRYRANSEQISSPKLSTTAPTASPAQQIPRSQPISVKGRQEHRKSSVSSDINSISPPAVQFAIGTPPTGRHRSTSGGSLSETPPPSAPCTWQVSPGGQTQSPLRRSGHSSPVLPSALTKLPTLGSPTLVVAPGSVGSLGSGSSSENNNQHPMLGPRAFTLPELGATGGLQSLLETNATGAETLMDREHNETLSKLNFVLALTDCIQEVADSRCAPLSALMAAGSNEQPQIPPHAPEHCKRAERLVLLVRALQLLSSGLNLASQQLRSGNLKPSSNVKNALLTMNSKYRSILFESKKLNGTGLLQKANAFNITADKILYDYALDMCQAAALDELLSNTKNCFERYNTAHILLHSLVQKCNHPQDKMLLNKYRDAVEKRLNILQQQGYIYVTDENS>g7003.t1 MATCH TO: RnrL Ribonucleoside diphosphate reductase large subunit [Drosophila melanogaster]MKSNKLFVLKRDGRKEEVHFDKITSRIQKLCYGLNMDFVDPVAITLRVINGLYCGVTTQELDNLAAETAATLTTNHGDYAILAARIAVSNLHKETKKVFSEVISDLHHYVNKETGRPSPMISEFHYNVVQKNAERLNSSIIYDRDFGYNYFGFKTLERSYLLKMNGKIVERPQHMLMRVAIGIHGEDIDSAIETYNLLSERYFTHASPTLFAAATNRPQLSSCFLLTMVADSIEGIFKSVEQCAMISKSAGGIGINVHCIRAKGTSIDGTNGTSNGLVPMLRVFNNVARYVDQGGGKRPGAFAIYLEPWHSDIFEFLDLKKNTGKEEHRARDLFYALWIPDLFMKRVEENGDWSLMCPHKCPGLHDVWGEEFEELYTKYEKEGKANRTVKAQALWFAIIEAQVETGTPYMLYKDACNRKSNQQNIGTIKCSNLCTEIVEYSAPDEIAVCNLASIALNMYVTPEKTFDFKKLKNVTKVITRNLNKIIDINYYPLPEAKKSNLRHRPIGIGVQGFADTLILMRYPYESEEAFLLNQQIFETIYYGALEASCELAEKYGPYETYPGSPVSKGILQYDMWNKIPTDLWDWAALKEKIKRHGVRNSLLLAPMPTASTAQILGNNESFEPYTSNIYTRRVLSGEFNVVNHHLLKDLTELDLWDDDMKNKIISNRGSIQSIEDIPKHIRDLYKTVWEISVKSTIKMAADRGAFIDQSQSFNIHVAEPNYGKLTSIHFYSWKAGLKTGMYYLRTKPAANAIQFTVDRGQHVTAHNESQQTNGDHDESAHNVSSSSFNNEREEREKRMAEMVCSLENKEACMSCGS>g8603.t1 MATCH TO: Pi3K59F Phosphotidylinositol 3 kinase 59F [Drosophila melanogaster]MDQPNDYFRYIYSSSLNELIQIKVGTLEGKKRQPDYEKLLEDPILRFSGLYSEECPSFQVRLQIFNQGRPYCLPVTTSYKYFTKRWSWNEWVTLPLQFSHLPRTAMLVLTILDCAGAGQTSVIGGTSISLFGKNGMFRQGMYDLRVWLGVEGDGSYPSKTPGKGKDSSKSQMQRLGKLAKKHRNGQIQKVDWLDRLTFREIELINEREKRMSDYMYLMIEFPTVVVDDCYTYSIVYFEPEGDKNYKFLSKPKLVTVPDSEILQENLVERKHHRLARSERSRISDRDAKPTASIRDQLHTIVYRYPPTCVLNSEEQDLLWKFRFYLCSHKKALTKFLKCINWETPAEVQQALILLSKWAPMDVEDALELLSPAFKHPQVRKYAISRLAQAPDEDLLLYLLQLVQALKYENFSDIMESHKRFFPDRDIVRSLDDNSTLFDQSSLCSELSAGGNQTTGGSSSSNLQATQRNNGGRTNIQIERSSTAPMAISVNSSLADENETSIGSLTSDVSNTLMSDTNTTNLQYSTMANSATNLATFLIQRACKNPTLANYLYWYLSIEVEDQESIRKQDEKVHEMYDMVLKMFMKVLENGNFNLRGIYYNLKKQQRFIDELVRLVKMVAKETGNRNKKTEKFQKLLSETDTFKISFTNIDPIPFPLDPDIYITKIVPSKTSLFKSALMPAKLTFVTSIASHEYVAIFKHGDDLRQDQLILQMITLMDKLLRRENLDLKLTPYKVLATSSKHGFLQYIESYTVADVLAREGSIHNFFRKHNPCENGPYGISPETMDTYIKSCAGYCVITYLLGVGDRHLDNLLLTTNGKLFHIDFGYILGRDPKPMPAPMKLSKEMVEAMGGVSSEHHHEFRKQCYTAYLHLRRHANVVLNLFSLMVDASVPDIALEPDKAVKKVEENLQLGLTDEEAVQHLQGLLDISITAVMPALVEQIHKLAQYWRK>g13135.t1 MATCH TO: fwe flower [Drosophila melanogaster]MSFAEKFTNLMARPNQQPGPADDQPWYLRYGVRILGIVGAFFCILFGLFNALSLLVLNVSCLIGGILQVVVGFLVMALEAPCCFFCIDYVGQMADMADARPMWNRAALYCVISIFPVFLCFGLGSLFPCALVFATGVIYGMMSLGKKASAADMRQAAVNSGIAGGTTTNDRAGIINNAQPFSFTGAVGTDSNV>g13135.t2 MATCH TO: fwe flower [Drosophila melanogaster]MSFAEKFTNLMARPNQQPGPADDQPWYLRYGVRILGIVGAFFCILFGLFNALSLLVLNVSCLIGGILQVVVGFLVMALEAPCCFFCIDYVGQMADMADARPMWNRAALYCVISIFPVFLCFGLGSLFPCALVFATGVIYGMMSLGKKASREDMAAAATSPTQMQPGGEGQMQMSTDQHITLMEDPDVWRPT>g13135.t3 MATCH TO: fwe flower [Drosophila melanogaster]MMPTNLQLNHLKLKLNFASLQHLHSSNFVNELTKRASAADMRQAAVNSGIAGGTTTNDRAGIINNAQPFSFTGAVGTDSNV>g78.t1 MATCH TO: fwe flower [Drosophila melanogaster]MSFAEKFTNLMARPNQQPGPADDQPWYLRYGVRILGIVGAFFCILFGLFNALSLLVLNVSCLIGGILQVVVGFLVMALEAPCCFFCIDYVGQMADMPMPD>g18882.t1 MATCH TO: morgue modifier of rpr and grim, ubiquitously expressed [Drosophila melanogaster]MASCMSEKQLQQNNNETMDTSDDDDDGLKDTELEDEPDKTQIQHTEENFNFPNSSTCWVCNGYYGPNFGEPLCGTCHAFLYNTEPTEELETTISDDEDSGNDEPPFKDKTEENDEDDEPEDEVDEVEDDRDVNDEDVEDVVDDVELEVDNDMIELPAAEEGANLEIDNAERILPPALPLDRPRPSAPRSLPQLMELLSEARANASEMATGAVTPAIRPNNILNLPVEIMVIIFSYLDDMSMWKASEVCKQWHNIIAQNTSQQMWRKYFKERWPLFQSLVDVQDWMKLYGSLMSSCFCRTCLLQMALKTPPRGRQNAIRANFLRNDFSLLNSYATEGIEAIPLDKQNTYWQASILGPPGSPYEGGKFFIFIFFPERYPMIPPTVRFLTKILHPNVSRHGDVGIDIFQHNWSLALNVSKVLLSVQSLLTDPYTEVCMEPDLGYMYEHNRPRFESLARSWTWKFAMFELIPPR>g868.t1 MATCH TO: N Notch [Drosophila melanogaster]MWLLVKNHTFAKSDIFWIKYLTLFLLIFSILQGISAAGSSCLSVGCKNGGTCITKTNGGSYCNCSSKFVGDYCEYPNPCLTGPGRCQNGGTCQVSYRNDRLGISCICPIGYTESLCEIKVPNACDSSPCHHGGTCNLKSLEDYTCACVNGYTGKHCETKNICATSPCRNGGTCTSVSGGTSYKCICPTGFKGATCIDDVEECNNNPCKHGGTCLNTHGSYQCMCPAGYTGKNCESKYVPCSPSPCQNGGTCRSTGLTYECKCPEGYQGKNCEQNIDDCPGHLCQNGGTCIDGVNSYHCACPPNYTGENCEKDVDECAIRPSVCQNGATCTNSQGSYSCICVNGWTGPDCSENIDDCVAAACFYGATCIDGVGSFYCRCTPGKTGLLCHLDDACTSNPCHADAICDTSPINGSYTCSCATGYKGVDCSEDIDECDQGSPCEHNGVCVNTPGSFRCNCSQGFTGPRCETNINECESHPCQNEGSCLDDPGTFRCVCMPGFTGTQCEIDINECQSNPCLNGGICNDMINGFKCSCALGFTGSRCQINIDDCQSQPCRNNGICRDSIAGYTCQCPPGYTGLSCEININDCDSNPCHRGKCIDGDNRFTCVCDPGFTGYLCQTQINECESNPCQYGGHCVDRVGSYACHCLPGTSGKDCEINVNECHSNPCNNGATCIDGINKYTCQCVPGFTGVHCEININECASNPCANNGVCMDLVNGYKCECPRGFYDPRCLSDVDECASNPCINGGRCEDGINEFICHCPPGYGGKRCENDIDECSSNPCQHGGFCVDELNAFKCQCMPGYTGLKCETNIDDCINNPCANGGTCIDKVNGYKCVCKVPYTGQDCESKLDPCATNRCRNDARCTPSPNFLDFSCTCKLGYTGRYCDEDIDECKLSTPCRNGATCHNVPGSYRCICSKGYEGHDCAINTDDCALFPCQNGGTCLDGIGDYTCLCVDGFDGKHCETDINECLSMPCQNGATCRQYVNSYTCTCPLGFSGINCQTNDEDCTESSCMNGGTCIDGINSYNCSCLPGYTGSNCQYKINKCDSQPCQNGATCHENGDEYTCHCSYGYTGKQCTDYVDWCTKSPCENGATCTQSKNQFNCRCAPGWTGKLCDVEMVSCSDAAIRKGVSLEQLCNNGTCKEHGNIHRCYCKQGYTGSYCQQEINECESQPCLNGGTCRDLIGSYACVCRKGFQGQNCELNIDDCSPNPCQNGGTCHDLVNTFSCSCPPGTAGLICEVNENDCKRGSCHNNGTCIDRVGGFECACPPGFVGSRCEGDINECLSNPCSNAGTLDCVQLVNNYHCNCKPGYMGRHCENKVDFCANSPCQNGGICSPKQGGHHCLCTEGFFGKNCEFSGHDCDSNPCQAGSCIIDDGGGYRCECPPGTEGRHCERDTMDECSPNPCLQGAPCDNLLGDFVCFCPPKWSGGLNDKYAKDFTYQQALCVKRGCEEKKGNHVCDPECNTYACNFDGNDCSLGINPWVNCTAKIQCWRVFMDGKCNEECNNAACLFDGRDCEKKLQPCNPVYDAYCQKHYANGFCDYGCNNAECNWDGLDCENAAEPPNLADGAISVILLMDLKQFREQQVVFLREMGHHLRTTVRVKKDSMGNDMIFSWKGTQPINDIQNTEFGRKNKILFSEHIGQTGIQVYLEIDNRKCTECFYSASEAAEFLAATASKYTLSANFPIYSVRGVTNPGEDVMESPTNVKYVTLGIILVVLAFALFGVLVTTQRKRAAGITWFPEGFRTPTINPQRRRRDPTGQEMRNLNKNPSMACMSNDVNMNSGHMGHAPQWSDDESDMPQPKRLRNDHGGYASDHTMVTDYEEADNRVWSQAHLDVADVRSSIMTPPAHQDGKHDVDVRGPCGMTPLMVAAVRGGGIDTGEDIEQSDDATAQVISDLLAQGAELNATMDKTGETSLHLAARYARADAAKRLLDAGADANCQDNTGRTPLHAAVAADAMGVFQILLRNRATNLNARMHDGTTPLILAARLAIEGMVEDLITADADINAADNSGKTALHWAAAVNNTEAVNILLMHHANRDAQDDKDETPLFLAAREGSYEACKALLDNFANREITDHMDRLPRDVASERLHHDIVRLLDEHVPRSPQMIAIAPQTMIASPPPGSQPQMITQPTVIAAGKQPSKAAQAKAAKKAKLLEGSPDGLLDNGGSLRRKPSSKKGLGQASKKGNNSSSLTASQLLNGLAGGQTVQNPGSVENQQQQLLDAALSSVDSPPPTALTSQPSPYDTTSMYSNALNGGGGQGTNHHNDLLGSNNVKHPPSYEDCVKNAQSMQSLVPQQSHDMIKMENYGYSMGSPFHQELMNGNQNGNNGLNGNNLLGGHEQGLSPPYSNQSPPHSVQSNMALSPHAYLGSPSPAKSRPSLPTSPTHMQAMRHATHQKQFCGSNLNTLLGGSGASALQSQNSPNSMNFQTPSSQNSPVSLGIISPTGSDMGIMMANNQQQQQQQQQQQHQQQQQQQQQMHQQQQQQQQQQQQQQNKNSAIMQTMQQQQQQQQQMGGNNGGPGMEFSSAGLDLNSFCGSPDSFHSGQMNPPSIQSSMSASSPSTANMLSPSSQHNQAFYQYLTPPSQHSGSHTPQHMVQTLDSYPTPSPESPGHWSSSSPRSDWSEGVQSPAANNLYISGGHQANKGSEAIYI>g5337.t1 MATCH TO: Cbl Cbl proto-oncogene ortholog [Drosophila melanogaster]MATSRTSRIQQSKNISSIFSKLHGAFSEACGPQRLSTDKKTLEKTWKLMDKVVKLCQQPKMNLKNSPPFILDILPDTYQRLRLIYSKNEDQMHILHNNEHFNVFINNLMRKCKQAIKLFKEGKEKMFDENSHYRRNLTKLSLVFSHMLSELKAIFPNGVFAGDQFRITKADAADFWKSNFGNSTLVPWKIFRQELNKVHPISSGLEAMALKTTIDLTCNDYISNFEFDVFTRLFQPWATLLRNWQILAVTHPGYVAFLTYDEVKARLQRYIHKAGSYVFRLSCTRLGQWAIGYVTADGEILQTIPQNKSLCQALLDGHREGFYLYPDGQAYNPDLSSAVQSPTEDHITVTQEQYELYCEMGSTFQLCKICAENDKDIRIEPCGHLLCTPCLTSWQVDSEGQGCPFCRAEIKGTEQIVVDAFDPRKQHNRSATNGRHQLTNDDDDTEDTGEFNIATSSLHALSNNMTSSTHSSDKHSPHTSPRFSRRSTTPSLMAVQNDLYAGHIPSLSLANSSVNYITAASAITGVHPPSAPPMNVVNGSNNTTNTISSSASASVIIPTAAASASNSAVATTPQSSQKSTNRMSAPLMGTNAATVIPGYAGKMSGKSENNDNSNSSYAILQNLQESQAIIAKEAQKREANSAAVASVAPPLPPRKSSPGAENSSSPMASNVILPSNGLVNGGGVIQKIPCIIPQTSKSVDNIVGSLGDICVPKTTAPPIPPHNTNSNVDTLVDELRVQRLSASSNTNTLLDDDIVGPAETIMGVIDTRPLEARSSTSNYTQLCTTTTTTTNSSNVSLRNEFKCQNEKLIEIPKQQIQIQSQTQTQQLQQQQHQHQKQQQQQQPLLYENVSINQKGDCSVPYENINLEYIARLMNEGYSKENAITALGISRNNIEMACDILREFVAKNSV>g11228.t1 MATCH TO: ntc nutcracker [Drosophila melanogaster]FKDKNINETNVVKRHCIDSSPASSSTFPLECVNLECLLLSDSSIECGVPVHVQTLLQNYDNLQLTQAEILLLLVYIVALESGFVAATHYAENKLCLQHLSATSSFHAKNILYLSHHKPKFLVTDDKTRFSLKLRTLIEMEDETTAALEDNLSALLTAMVTGDFLIITLTPIPTTNAKGFSLALSMPRYVLSMPIKNKPLYQRFRKLPELSYILREQLFGPMRCQQLSLMECCIYPSFADLPAELYKHIFKYLNKNQLNILANVNKSLYNLIKCIGFQK>g19655.t1 MATCH TO: ntc nutcracker [Drosophila melanogaster]MSQHSTVSTTTFTNTTTTTTSTTAVTTNEESSSAKSGSSSILTGSLKKQKHKDVTPAEVPLTDDFVLHIPPDKFKDKNINETNVVKRHCIDSSPASSSTFPLECVNLECLLLSDSSIECGVPVHVQTLLQNYDNSQLTQAEILLLLVYIVALESGFVAATHYAENKLCLQHLSATSSFHAKNILYLSHHKPKFLVTDDKTRFSLKLRTLIEMEDETTAALEDNLSALLTAMVTGDFLIVTLTPIPTTNAKGFSLALSMPRYVLSMPIKNKPLYQRFRKLSELSYILREQLFGPMRCQQLSLMECCIYPSFADLPAELYKHIFKYLNKNQLNILANVNKSLYNLIKCIGFQK>g14846.t1 MATCH TO: pnut peanut [Drosophila melanogaster]MQNSPRTNGAISSLPSTLAQLALRDKQPAPALPHTLPPSVVGGSNVLSKASALQQANGGGGGGVGNDVNNVGHNDSNKLSTELQEKEQQDNKQQQQQLTQPKQKPPLPVRNKTMEIAGYVGFANLPNQVYRKAVKRGFEFTLMVVGASGLGKSTLINSMFLSDIYNSEQYPGPSLRKKKTVAVEATKVLLKENGVNLTLTVVDTPGFGDSVDNTNCWVPILEYVDSKYEEYLTAESRVYRKQIPDNRVHCCLYFIGPTGHGLRPLDIACMQSLSDKVNLVPVIAKADTLTPDEVHLFKKQILNEIAQHKIKIYDFPSTLEDVAEDSKTTQNLRSRVPFAVVGANTIVELADGKKVRGRRYPWGLVEVENLSHCDFIALRNMVIRTHLQDLKDVTNNVHYENYRCRKLSELGLVDGKARLSNKNPLTQMEEEKREHELKMKKMEAEMEQVFDMKVKEKMQKLKDSELELARRHEERKKALELQIRELEEKRREFERERKEWEDANHITLEELKRRSLGANSSSDNVDGKKEKKKKGLF>g11763.t1 MATCH TO: wts warts [Drosophila melanogaster]MHQAGGKSGRPKNKYTAEALETIKQDLTRFQVQKDNGIQQNFNHLRYSAVNGRADAPEYHHAKAPMEPPPSASSSPAIPTSNQLNGHVPKVLPQSIIPPKLNRKASIERELPMNYLRCSPALDSGAGSSRSDSPHSHQQITCASRASTGQYSPSPSSFSDVAPPAPPPRNPTSANSATPPPPPPSNQHYKRRSPATTRPAAIAPNPTRGTSPVIPQNGIKAQQQLSQQMKALSLYQAGGASAVEPPPPYPLNNVMVPTSAPPPSYSASMQSRQSPTQSQSDYRKSPSSGIYSATSAGSPSPITVTSSAIPPPIQPSSVARPQPRVYPTRTQQPIIMQSVKSTQVQKPVLQTAVAPQSPVTASACNSPVHILSAPPSYPQKSPAIVQQQQQQQQQSPLLAGGVQTVVSKPTPPTPTTPPLIANALNGTVMTKSPCVEPPSYAKSMQAKAAQHHQQQQQQQQVAAIQQQQQQQLQAQLRAAAALAQQKPPVTVPLVGGRQLPPPPPYQSTRTATAVPPPSSSPHDLNSNNNNMLDSRSNSGGGGGGNQITNSNLTTTPPIPPSKNMNNNNGGSNGSGGESVASSSSGAAASCSSKCTPPDNVKKIKHASPIPERKKVSKEKEEERKECRIRQYSPQAFKFYMEQHIENVLKSYKQRTFRKHQLEKEMLKIGLSTQTQVEMRKMLNQKESNYIRLKRAKMDKSMFVKIKPIGVGAFGEVTLVRKIDTTNHLYAMKTLRKADVLKRNQVAHVKAERDILAEADNNWVVKLYYSFQDKDNLYFVMDYIPGGDLMSLLIKKGIFEEHLARFYIAELTCAVESVHKMGFIHRDIKPDNILIDRDGHIKLTDFGLCTGFRWTHNSKYYQENGNHARQDSMEPWEDFSDNGTKPTVLERRRMRDHQRVLAHSLVGTPNYIAPEVLERSGYTQLCDWWSVGVILYEMLVGQPPFLANSPVETQQKVINWEKTLHIPPQAKLSREATDLILRLCSSADKRLGKKADEVKAHDFFKGIDFADMRRQNAPYIPKIEHPTDTSNFDPIDPEKLRSDSNMSGDEFNDTDKPFHGFFEFTFRRFFDDKLNPDVMDDQSPVYV>g19935.t1 MATCH TO: Abd-B Abdominal B [Drosophila melanogaster]MVRFVKVMTANTVYFRQHVHHIEIIGSHAAAGAWWGPHHHATPHPSTTAATAALNHHQTATVTTHHHHQQLHHPAHHTHPHPHAHLHPHHQPHLQHHHLLQQQQHNSSTTGLASSNASSASSLLGTETALQQQQSSQQQSSTQPTTHSTPTHAVMYEDPPPVPIVQQQQQQQQIHLPHQQQQQQQANSQQQQTHNNVETTPVGCLSPTETPSGPTSQSQQHLTSPHHQQQQQQQSNTVPSTAPSSIQQQQQQQQQQQNTAVASGQTQIVTPTAASVSPSSVSSQPPDMSLSLAPLHIPAIRPGFEADAAGAAVKRHPHPGWPYESDGFSSAQYHHASPYYLERDRKPVFYGYPETQFQYWNYREQPPSAAAAAYMSANDDRHANVSASATARQSVEGTSQSSYETPTYSSPSGLRAYPSEAYSSTGGSGGLSVGAVGPCTPTNALHEWTGQVSVRKKRKPYSKFQTLELEKEFLYNAYVSKQKRWELARNLNLTERQVKIWFQNRRMKNKKNSQRQSNQQNNNSSSNSNHNHSQAAQQHHNNHHLGLGLSMGHHATKMHQ>g3439.t1 MATCH TO: lkb1 lkb1 [Drosophila melanogaster]MTVTSSHVTMEVQAKAHQPANHQGTSGTQMISNPSPSFSKNIENYRNEGEKTKAAGGENSTLILTNTKFHLDTYPDIVDTICEVSSCQGIFVDGVNAENQNLSEQQRYQNQVTWLDEDNIDTLDLATLDIGNMFNRVDSAEIIYQQKKKNIKMVGKYVMGDVLGEGSYGKVKEVLDSENLCRRAVKILTKRKLRRIPNGEQNVQREIQLLKQLKHKNVVALLDVLYNDEKQKMYLIMEYCVGGLQEMLDSAPEKKIPLFQAHRYFRQLLNGLEYLHGMRVIHKDIKPGNLLLTLDETLKISDFGVAEQLELFAADDTCTTGQGSPAFQPPEIANGLESFSGTKVDIWSSGVTLYNLCTGQYPFEGDNIYRLLENIGRGQWEAPDWLYKLDSNLALLILGMLQSDPNKRYTIEQIRKDSWFISAPEEKGPPIPIPPLKNDKYRRSTVLPYLDAYHYETERDLEEVYFTEHDLNQELARKAAAAAAEIKAQQHNSFSAVGTSSSGFHTSCSNSASHHTKEKKTSSLKRRAKKLTSCISVRKLSNCRPS>g5482.t1 MATCH TO: CG14806  [Drosophila melanogaster]MFCSTMITVEKMFFRRYKVKQKSQESRKLLCTGVYKKLEKFHDKPDPHLIKHDYIGPPDKESNLRPFVRCIPKNESDLEKRLRYKRIEIEEWNQSFWSRHNRRFYEEKNEFVRLHKSSGTQDISADKMSEFYKSFLDKNKKVHILYNISWSSTFRLYCSPYYWYAPVLKVFPGNGSAQCPEPLAQHVNTIKLHFLFELVSSQDLFWTETLKGVIGKLCNKVIMRHRRPTQHHHQSEHLRDSSMNECRPWRPPTAHFELEQILPRQQE>g16639.t1 MATCH TO: Mer Merlin [Drosophila melanogaster]MSPFRSKKPRVFPVKVITFDSELEFELEQRASGQDLFDLVCRTIGLREYWYFGLQYVDTRCNVTWLKMDKKVKDQRIQLQPNGFYVFSFYAKYFPENVSEELIQEITQHLFFLQVKQSILSMDIYCRPEASVLLASYAVHVQYGPYDSETYKDGMLQGVDLLPKGVTDQYQMTPKMWEERIKTWYMDHEPMTRDEVEMEYLKIAQDLDMYGVNYFPITNKNKTKLWLGVTAVGLNIYDYHNKLTPKTTFQWNEIRHVSFDDKKFTIRLVDAKVSSFIFYSQDLHINKMILDLCKGNHDLYMRRRKPDTMEIQQMKAQAKEEKQRRQIERTKFLREKKLREKAEQDRYELESRLERLQEDMRMASDALRRSEETKELYFEKSRVNEEQMQLTECKANHFKSEMDRLRERQMKIEREKMELEKKIRDADFYVHQLTVEKDKREAEAEKLKKELICAKLAEREATARLLNFLNCGRKNSQDSVLPPNITVNSSSSSSNNIAASVSTPSLTNSNSTADINESTGGVELTKNDLNNLNQVRVGLGVGEVADDDSENDDFETKEFILTDSEMEQITNEIGRLEYLKKHKQVQNQLQTLRSEIELLKIEENQTNLDILSEAQLKAGETKYSTLKKLKSGSTKARVAFFEEL>g4014.t1 MATCH TO: hpo hippo [Drosophila melanogaster]MSSKSELKKLSEESLLQPPEKVFDIICKLGEGSYGSVYKALHKESSSIVAIKLVPVESDLHDIIKEISIMQQCDSPYVVRYYGSYFKQYDLWICMEYCGAGSVSDIMRLRKKTLTEDEIATILSDTLKGLVYLHLRRKIHRDIKAGNILLNTEGYAKLADFGVAGQLTDTMAKRNTVIGTPFWMAPEVIEEIGYDCVADIWSLGITALEMAEGKPPYGDIHPMRAIFMIPQKPPPSFREPDRWSTEFIDFVSRCLVKNPEERATASDLLDHEFIRNAKQRGILKPMIEETCAIREQQRTARSAGGSVQGSQAKSLATQQEDLLHEEEQEFPAETVKTFIDDPGTLVPEKFGEYQQTSNASDATMISHPEDTGTLCPGKMTSAGGVAANKSSDGGDIAAVDSGTMVELESNLGTMVINSDSDDSSTAKRNDFVKPRYRPQFLDHFERKNPTTDVSDKITEYSPAAAHVAALAAQANNVNNHNLNNHQHPPLSNANSNDTNWENNMEMHFQQISAINQYGLQQHQQHLQQQQLQQQQQAAANAAAAAAAAAYYGVNPMVNDQHLLALNQQQQQQKQQQQQHQMQQPPAYPQPQQPQPPAHHPHHHLHTQSHTYVDGEFEFLKFLTFDDLNQRLNNIDSEMEKEIEELNKKYNAKRQPIVDAMNAKRKRQQNINNNLIKI>g449.t1 MATCH TO: ik2 IkappaB kinase-like 2 also I-kappaB kinase ε(CG2615, FBgn0086657) [Drosophila melanogaster]MSFLRGSLNYVWCTTSVLGKGATGSVFQGVNKITGESVAVKTFNPYSHMRPPDVQMREFEALKKVNHENIVKLLAIEEDQEGRGKVIVMELCTGGSLFNILDDPENSYGLPEHEFLLVLEHLCAGMKHLRDNKLVHRDLKPGNIMKFISEDGQTIYKLTDFGAARELEDNQPFASLYGTEEYLHPDLYERAVLRKSINRSFTANVDLWSIGVTLYHVATGNLPFRPYGGRKNRETMHQITTKKASGVISGTQLSENGPIEWSSTLPPHCHLSEGLKTLVTPLLAGLLEENREKTWSFDRFFQEVTLILRKRVIHVFFSNRTSSVEIFLEPEEQIEHFRERILMQTKVPLEKQILLFNNEHLEKRINLHTTANNFPKTTPENPIFLYSNDDNNVQLPHQLDLPKFPVFPSNVSVENDASLAKAACSVGHECKRRVDTFTSIDILIKKAVEQFIETLTTTITLLVKKTKNFDKLLTTEIDFADAVQSMAKMTKGDQELKQILQKLENLKTDFDGAADVIMQLHANFVVEDQLNEQWSSSMHGKKCPCRTRASAQAKYLVERLRDSWQHLLRDRATRTLTYNDEQFHALEKIKVDRNGKRIKSLLMEDVKPSVAQMAECLADWYKLAQTVYLKSEILEKDVRECERTLNSIRDDLYRVKMDLQKDMDAKLVNNSNQLSKIEQKSRLLFMQQQQQEILGIMKQNRQLISMLQDLGIRLPTTANPLEF>g4848.t1 MATCH TO: PDCD-5 Programmed Cell Death 5 [Drosophila melanogaster]MEDSELDAIRAQRMAQMQAQYGGSGGNAAEKQKAQEEQMRQQEEMKNSILSQVLDQQARARLNTLKISKPEKAAMFENMVIRMAQSGQLRGKLDDAQFVNILESVNAQMPQSKSTVKYDRRRAAIDSDDDDDYGC>g19924.t1 MATCH TO: Dfd Deformed [Drosophila melanogaster]MSSFLMGYHPHAPHHVQSSMSMNNGLDPKFPPVADDYHQYNSHYSMTASTGHMVPGGGSGAGGMPTHPHATHPADMVSDYMAAAAHHHSATSHHHPHSHTNSALSGHHQTSSYTNYASTTPTHHHHHAHHQNLGYYNHHAAAAAAAAAVHHTPDYLSAGPALHNDPSVTSLGTTNTYAPALTSPNGGGGNPITTNGYYGGYYGTNGSVGSTHSQGHSPHSQMMDMPLQCSSTEPPSNTALGLQELGLKLEKRIEEAVPAGQQLQELGMRLRCDDEGSENDDMLEEDRLMLDRSPDELGSNGLDDDLLDSDTDDDMMAETTDGERIIYPWMKKIHVAGVANGSYQPGMEPKRQRTAYTRHQILELEKEFHYNRYLTRRRRIEIAHTLVLSERQIKIWFQNRRMKWKKDNKLPNTKNVRKKVDANGNPITPVAKKPKRVSAKKQAQQQQQQQQQQQSQQQPVINECMRSDSLESMGDVNTSLNNQPYIPAAGDTASLMNPQQIPSNSHVNVNTSSGNINNNNNSNSNNNNNNNNSLHINNNNNNTGNNNTNPMQSNLHSQHQQQGHVQHAQDLMANLQQHIKQDYDLTTL>g17952.t1 MATCH TO: ex expanded [Drosophila melanogaster]MRAFCTVSAPLEVCASSAEQLSPGSRFLALRLLGNQQPKTLYFLVDAKSRVREVYTQTCLHFATQGMLDTELFGLAVLIDGEYMFADPESKLSKYGPKSWRSSHTHGLDANGRPLLELHFRVQFYIESPFMLKDETTRHNYYLQLKYNALQRDIPREYAEQSMILLAGLALQADLGDAPCSGINNSNSSSSTGSNGLQMGESKLASDLGESKLDSAKGEQKVSKTLSSTSSASSTTGVATTTLPKISKRANMVNERVLRLSSYMASTRENVMGMDGLKRVLGAAAKTPLSPSSSSSSISRGPSNASTNASVSTTASSCNEYFRLEDYLPEHLRTAWAASALRACHRENRGLSQADAELHYIQQACLVHECINAHTFRMRMSKSEIGPGNSWFVVYAKGIKIFGASTGPDGQPQQITFLWPNITKLSFERKKFEIRSGESKITLYATSDEKNKMLLTLCKETHQFSMKIAARLKEVIKREEEESNCLHACYVYSRSLHLPTYKNKNDQRISVISSTSSNTTSGIVSDRVHSEDELEIMINSPPAPIAAPSTESLALAHLLDRPSVSRQTSSVGQVSLKDLEEQLAALTVRQQQQSQRNSPTELSNTSSTGNSTSTSGSSIPTSKGQRNNTDSSTDSPSSQHNIGSQCSSTCSTVVVASSTNDNNTSLATLAVRSSLTTTDSNHQRKNSTSSSLELGFSHTAQNSTLSEPESSCIDHDFISTSRDETESVSGVYTLVHAVAPTETSGVYTMHSSEMTGQSSEIAESEKSSHYGIFQPSQSNISEEHMRLEHNKRQDSVDGGNYHGKRMKNIDFRLRSDSNISTTDSFRGDGSDPADMKHTLLSAEELTDLIVGRGSYPNRKTVSSTLDSDCDYVTLPLAMTGESYIQGHQDTAPTEDHVEDLISDLLPTDPPAPPKRVDSNMPNLTNIRTRSPPPYNARHHKTGLCGPPIRTTLSSTPPAPTTPINVPVIKTSTPNHVPLSLTDTKPLTKPMIIAKPPTPPIIQPPIIKRRDPPPYPAASKPRPTSLISVASSTSSLNQPGSNVFTNVGGSMTSLKSEEITARFITTRPQINILKAHTFMVNENAKPSYAAPTNCSSAASSTGSICSHHIPSHLSQHSVPNSNYVSGSQASLNLHHTITSSPTTPTQPSNLGHTSMAGHHHTTAAAGVLPNPMSIPMVSYSLHGGHKSMSSLHQQQQPPPPPPPYPAELKQPAPRTCVLLPVIKPRQYLPPPPPSLPRQPPPPPPTQLANLYSSPLARKQLELYQQQLYSDVDYVIYPLQDPAVSQQEYLDAKQGSILAAMAQTPPPPHAHHHHHPYLAAYHHAAASQAWEACKGHAIYRSTPYLPLALSTHSRYASTQNLSDSYVQLPSAYSPMYSPSMASICSSYEPPPPPPLHPAHLHAAAAAAAVVNPNPTPLFARSRSDDNILNTLDSLPKVKRLPPPPPPPYVDRRLKKPPMPAPTEKPPPIPSKPIPNMSGGASISIPNESINSCTNRSQLPPRKPITLSVPRNGAIHMTKTSSGAQWAGISSCCQTKSINTTSTTSSTNGSTSTTANTSTPNEFDIALLREKSKHLDLPLISALCNDRSLLKQTKVLVNPKNPKQTTLGNNAATATSTSPTLSGGSTENTGTLNKLPLSGLSLNNGTNTTLSSTASTSTTTASAAATATSSSKILSSTTVTAKTRKTSISHRHPNDKLPPLPMQLAEANNYVMDPAILKHKSYNSHT>g15951.t1 MATCH TO: Fadd Fas-associated death domain ortholog [Drosophila melanogaster]MGQPINFDTLKCIVANDQTVQDNLENLKSLFRSDISSVRNMMRIRTVADLLDCLERHDSLSPYNIEPLREVADLCGGALEDLVASYYVPNTPELHNEYHELRISYEMESRLQLSGLVNGITNNGGVEANINQGNSTRREPVFAHVLSDDKNAAIQKLIASDIGTDWRCFGRELEVCQGDLDNIEINYRDLKTRVFKLFQVFEENDAIDPKKHLHIIMKALEECRRKDLCRKIQKILSH>g15211.t1 MATCH TO: Der-1 Derlin-1 [Drosophila melanogaster]MTDAGQWYRQIPRFTRYWLTAAVTMSLLVRFGILPFNQMYLSRELVFSQLHLWRCITSLFVYPLVASTGFHFLVNCYFITQYSARLEKDQFGRSPADYLYLLLIVSILAILGGLLFNVPFLMDPLVVAVTYVWCHLNKEVIVNFWFGSRFKAIYLPWVLAGMELIFQGSIASLIGIFIGHFYYFLKFQYPQELGGNAILETPLILKQYLPDVSGGFGYFGVPPASRSNQRPGGAAEAAGIWGRGNTLGH>g1587.t1 MATCH TO: Traf4 TNF-receptor-associated factor 4 [Drosophila melanogaster]MVRSLAQWTKTLSFPSRLSPNRNSKDCSTLNATSPIPPPTPPRNKNSNCATSRSSSSTVSSNSGTSTHSSNNNNMPITELEQIIYPGPDPKQAIMGSLVFCIHHKQGCTWSDELRKLKAHLNVCKHDATQCPNKCGAQIPRIMMTDHLQYTCTMRRTKCEFCQSEFSGAGLEEHAGTCGQEPIYCEAKCGQRVLRGRMTLHKSKDCTKRLRRCIHCNREFSADTLALHVNQCPRAPAVCPQRCDVGPIARADMEAHLRDECKALAISCTFKEAGCRFKGPRHMLEAHLEANAASHLSLMVALSARQGQQIQMLKSAVSKLSINFTGTLLWKITDWSAKMSEAKGKDGLELVSPPFYTSQYGYKLQASMFLNGNGPGENTHVSVYIKVLPGEYDALLKWPFSHSITFTLFEQGAQAGQGGVAESFVPDPTWENFQRPSNEPDQLGFGFPRFIAHDLLYKRPFIKDDTVFLRVKVDPSKIVAV>g1588.t1 MATCH TO: Traf4 TNF-receptor-associated factor 4 [Drosophila melanogaster]MLTSTMSFNQKQILPLPPPPLPPSLSSTSSSQQQQQKQQSATSCLPTSHTTKIHSSCSNTNLNNIIGNNNNSNNNNINDSRTHLSAIAPTPCQRLSEMFRHSIADSSIYSESRENTYEEVSY>g17494.t1 MATCH TO: MED24 Mediator complex subunit 24 [Drosophila melanogaster]MLVLPRGVSGDVYNLSDCILQQALIGSTANPLVLNYLKHSLCAHLVSYSAVLRRISKYEYFERIYCITSLLDFLNSILDGVTCRTKTEESILPGAIVSLVNWLMQIFAMVATNYEMNREISAEQSYMLDQCCVVIEKLVKNQFLLAILYVGRQEDLEYYSKIRENYATIKTSLTNSNFTPSHPGIEKYLQQLAYIDIHHLEMKTLDVAQLPEPIAYCVQPLLAVEVLLNPCNDTSYYVAEMQMLQRLKKFSNTRLFYEIIRAGFVSLSNVVETSHDTMWGAFTFFKVPQIIKQLHALNRAPGEQNPPDYIPEVVEALEMLLEDNLLLDFMDSKCSCNIIEYLLNDWTKQHLVNDAHVKHFAAQREEVSMLLQKREAGNIPSIINFIIRAEVPLSGILKTLSADYNKVQEALLGVLCQVLVGNSFDLILSVATVEGRLKTFISRLIQCNENSKQMPGEVGKPSIIRSTLFDVSFLMLTSIVQTYGSDALISETGDSFFEKWVRIYMVERNKPKNYHNILALCDENIVDELLLSFSKPEVQLKNTNITWQEICLNLPGVLYHVLLAWEKETLSSADVKNILDNIKRRMFSFSVCAASFLCAYMHSVKKVRR>g17495.t1 MATCH TO: MED24 Mediator complex subunit 24 [Drosophila melanogaster]MIQQFLSPPGNEELSSQENFKERLGLSLQIIRKMQYDVHPTGNLKSRSLTLTQNLVSRNPLIDQFKDVWNTVLEHGWVPVRAAQVIESLLHAGGAAWLASRLVEEMLKCKYKKDMMKMMDIIFAILHLDIEKTTEALLTNVVPSIIHNRQGDDINEPQSYVLARLCVYCIISALEARSQNSPTQKKRSRSHDGDEHDLNNAAKMRKITADGSDNSCSNDFLSENSLLLSSSVSMHSNSLRETPSQLKESLQTAVQYIFKVFQQFVTTDELSPKIYFVYQFITLLVECGKERVRPVLKLLPPNLINNLIKVMLTDDINVGLITRLYDLRVNTGRQTAVSDLCLWRNIKLKQQSIQL>g15747.t1 MATCH TO: CG32202  [Drosophila melanogaster]MLLDDKQQKYKQFVDKLLQRCQEIQTENERHVLRINTIKKLIRRRVRDVELLKRRLDQHNDNWRSLPMVAPHPKRKIEQKRGPKPKNKDSNGNDAKEKKVRKQRVKKNGDEKLPVSSLAEVQDMLEQSRYQESQAQQLLMMQQRQMNLKNDQLL>g5935.t1 MATCH TO: Drice Death related ICE-like caspase [Drosophila melanogaster]MSYTIPNHADFIMTYTTIPGYTSWRTFVRQRVATCYETCNRNEYNRKKQTSCDVGTTRLLYFRIPEELLQREFTIYSQQFFQVLLDRTLDGQDQGEVLLNPHSAHVLITNNLFKSYDNTDLMFAEGQQRP>g16321.t1 MATCH TO: Drice Death related ICE-like caspase [Drosophila melanogaster]MATASEDQVGTFVERSTNTDDITDAFGSLGSSNSSSTNTYYANSSSSHHQQGAIAQLANKSFNTSSYNYTAIMPTDRHAVEYNMRHKNRGLALIFNHEHFMVSTLKSRSGTNVDCDNLARCLKQLDFDVRVYKDYTYHEIKHQIEWAASQDHSNNDCILVAILSHGEMGFIYAKDTQYKLDSIWSYFTAQHCRSLAGKPKLFFIQACQGDRLDPGITMKRMITQTDGDSSMSYKIPVHADFLIAYSTIPGFYSWRNTTRGSWFMQSLCAELAANGKRYDILTLLTFVCQRVAVDFESCTPDTPEMHQQKQIPCVTTMLTRILRFHDKNSAAPAGRV>g17258.t1 MATCH TO: Drice Death related ICE-like caspase [Drosophila melanogaster]MLRRPKNARCALASTDRSAMEYNMNHKQALIIIYHENFAHTKATLQTKKDCEDLRDALSKLEFAVSIYYDYTWSQINNSVREAAVRDHSQNDCHSQSIVIAILKHANKCPTLTGKPKLFFIEAPRGKMLDSGITMRGTETDGKGTSDLSYTIPTHATIHENTTPSNNENFDDFEAINGWFNRFKTRGNLHNVALKGESDSADSEAAEKFKCNEKRETKDICNYTFQSFQNSSNGSWFIQTLVAELNKNGKSRDLNPLLTFVNRRVGIEYLSGNPTDIEKDKKSKYLVLPQC>g14729.t1 MATCH TO: TER94 TER94 [Drosophila melanogaster]MADSKGEDLATAILKRKDRPNRLIVDEATNDDNSVVSLSQAKMDELQLFRGDTVILKGKRRKETVCIVLSDDACPDEKIRMNRVVRNNLCVHLSDVVSIHPCPDVKYGKRVRILPIDDTTEGVTGNLFEIYLKPYFLEAYRPIHMGDNFIVRAAMRPIEFKVVLTDPEPYCIVAPETVIFCDGDPIKREEEEESLNAVGYDDIGGCRKQLAQIKEMVELPLRHPSLFKAIGVKPPRGILMYGPPGTGKTLIARAVANETGAFFFLINGPEIMSKLAGESESNLRKAFEEAEKNSPAIIFIDEIDAIAPKRDKTHGEVERRIVSQLLTLMDGMKKSSHLIVMAATNRPNSIDPALRRFGRFDREIDIGIPDATGRLEVLRIHTKNMKLADDVDLEQIAAETHGHVGADLASLCSEAALQQIREKMDLIDLEDDKIDAEVLASLAVTMENFRYAMTKSSPSALRETVVEVPNTTWADIGGLENVKKELQELVQYPVEHPDKFLKFGMQPSRGVLFYGPPGCGKTLLAKAIANECQANFISVKGPELLTMWFGESEANVRDIFDKARSAAPCVLFFDELDSIAKARGGNVGDAGGAADRVINQILTEMDGMGAKKNVFIIGATNRPDIIDPAILRPGRLDQLIYIPLPDDKSREAILKANLRKSPLAKDVDLTYIAKVTQGFSGADLTEICQRACKLAIRQAIEAEIRREKERAENQNSAMDMDEDDPVPEITRAHFEEAMRFARRSVSDNDIRKYEMFAQTLQQSRGFGQNFRFPGQSSNQQGSGPNAPANPPGDNGDDDLYS>g3131.t1 MATCH TO: Dredd Death related ced-3/Nedd2-like caspase [Drosophila melanogaster]MSSSKQLNELEKLIEQEDLPFIETDLNFSQLVSLGFLLYADDSKSLHFILQKLIILSQQPPLGQGLKQLGKPASSDILLNYAKLNPSKWQQHIVEALAIIKAKKVLRKLGFNWQQLMHYYLPHVAEISVNIHPLLKALYAVCERLTIDQSYRLITYINDKYKQCQELRFYDSSHLEVFLLNWLTKDVITLGSKQMQDSNVQILIEYFKFNDMLELKDLLVFTINQNIDNNEDVHKNVLPNDNIPNTEENSKDSGVSMSHDNNNDSNCNKNDSVAKSSNIKLSEQPKQQSVKNLEEEHDKEHYAIKKQSAGYVLIINQSKFYYETDPKYKNLIPPITEAIPQRQGTNVDRDNLKQVFSSFGYKPIIFENLTHLEILHHIRETVKKSLLKDSLIICILSHGMEGVIFGSNSVPVSIDEIKTILTADTLNGKPKMLIIQACQRNICGNDMVKNSLVKEDFSPDAYADMLIALASMPGTEALRHTEHGSWFIESLCNCIKKLNKSKHMLDILTAVINDISRRRGDRNQVMLPFTSSTLRKEFFLPSN>g7359.t1 MATCH TO: HtrA2 HTRA2-related serine protease [Drosophila melanogaster]MALQYLHRFSLLTKNIAHSAALRTSVHVERYTLIGQQNDFSTNQQNHKNYNRNRQQRREWKTAALTGATLAGVGTLYWLKERNDLNVYVWPTVEAKTLPNLSGRRKQFNFIADVVDVCANSVVYIEIKDTRHFDYFSGQPVTASNGSGFIIESNGTILTNAHVVINKPHTMVQVRLHDGRVFPAVIEDVDSSSDLAIIRINCKDLPVMRLGESSTLRSGEWVVALGSPLALSNTVTAGVISSTQRPSQELGLRNRDINYLQTDAAITFGNSGGPLVNLDGEAIGVNSMKVTAGISFAIPIDYVKLFLEKVKERRKQGATGKPTQQAKRYMGITMLTLTNEILNELKNRSESLPSNLTHGVLVWKVIIGSPAHIGGLSPGDIVTHINKKEIKTSSDVYEALADNSKTLDITIFRGPKRLNLTITPEDP>g3861.t1 MATCH TO: Dronc Death regulator Nedd2-like caspase [Drosophila melanogaster]MEKNQREKILNNIDKLIDYTDFEQLCEACCNDELLSSTMITNIKRTTPSNQSVGPKALYDDTLERERHKQLFIKITKRGPQAFQKLHRIFVRLKYEDALKILFNAHEDHSLRSTYNIGGYRSTNNDNQSSDGDKIVDKITGQLSINNNNNVTLQEYDARLIQPKKTLLSDEHQEFDDDDHLLLSLFQKLSFKIYAYNNITFQVFTKLLVELLGSDKIKDIECFILFIRTKGKLHNNMQRMKFNDGSIVKIEQVHKYFYNNNCPQLLGRPKIFIFPYKEIHKRRVQTDGLGEVLRSPHVPQMSDVIDCYVANNGYMDYRDNNNFSCYIQNFVDVISERACDTSLDDMLKIIHTKVAEQSAISGQLQESSYENISQNKMLYLNPGISN>g3863.t1 MATCH TO: Dronc Death regulator Nedd2-like caspase [Drosophila melanogaster]MDDNDRELIIKNIDSLVNLTDFKTLHQYCVLYGLLTDVMVHNIYHADLEKLSTDSCDEIAITRERHKRLFLKITKRGPDALKKLQLIFKDLKYKEASKLLFGTVHSMISISLSKPTLTPTSGENDDHEEDNDLENIQSGNVDDRDGIMRRQFSRTFSNDNEISLDQYNGVIKPKKYVNVRKATKISKHPSIETYPMESKSNRGVFFMVNIVDFTTEPKRKGADEDTHSLLSLFKQLDFKLYAYTNISQKEFFERLDMLLKSDVIKDTECFVMALMTHGILREGIQWVSFNDGSVAKVEDIEKRFYHENCPHLMHKPKIFIFPFCRGDIPDRGVVIPNKTQTDSIGTSAPITNNVCGTLSDVMICYATVAGFESHRDVEDGSWYVQKFVDTMAEHACDIPFEEMLKIVQSDISKLRTENNELQTANYANIGFNKILYFNPGVFRE>g595.t1 MATCH TO: klhl10  [Drosophila melanogaster]MQNYDHNEENQDEGVEMPHTDSEVMDDIEDEGNDEYVVAPKRLKISVSAQALKTLHDLRCSNLLCDAVISVSDATFNVHRAIMSSCSSYFRAQFTGFNSNEVKCLKEENHLVHIPGMSGNIMEQIIQYAYLRKCTINDDNVHELLISADYVGIIGLVELCKQYLAQMLTPENCVSIMGFARFRFLDDLYAKARNYLLRYFIEVAAKNNDIYELNLNDFYDIISDDELNTREEDNVWKLCIKWIEHDPDNRKQHVAKLMQGVRLGLMTPKCFMEEVKEHPYVMQSEEAKPLIVETFKFMYDLDIMNLSSGELTTPALAMPRLPHEVIFAIGGWSGGTSKGCIETYDTRADRWVNIPADDPAGPRAYHGTAVIGYKIYTIGGYDGVEYFNTCRVFDAVKKIWKEVAPMHSRRCYVSVAELNGLIYAIGGYDGHNRLNTVERYNPKTNQWTVITPMNMQRSDASACTLKGKIYATGGFNGQECLDSAEFYDPLTNVWTRITNMNHRRSGVSCVAFRGQVYVIGGFNGTARLSTGERYDPDAQTWTFIREMNHSRSNFGLEIIDDMIFAIGGFNGVSTISHTECYAPETDEWMEATDMNVVRSALTANNVAGLPNKRDYIHKERDRLMEERRQRLLASAMTREDNNTTSYMSVETQEAAMDDYETPNEDEDADDEDDVLQDLPQIPQGPQAFNDIMNNQEQQRRFRIQQLRNQRVGSHQEIRRR>g18935.t1 MATCH TO: Den1 Deneddylase 1 [Drosophila melanogaster]MSSYTHADPIALSFNETCLRMSDIQLLQGPHWLNDQIISFYYEYLERVKYKNNPDLLFVPPEVTQCMKFSDDGELETLLNQLEAPKKPFIFFVLNDNNTTQAGGTHWSLLVFSRPEKSFFHFDSWGNNNSHASHQFVQRIKEALNCRICQIKPIRCLQQANGYDCGIHVICMTDNIADNVNRYECVEGIGPLHQDIISAKRTDLLKLIQSLGGKI>g4871.t1 MATCH TO: kibra kibra ortholog [Drosophila melanogaster]MPKLQQQQQLPHQQQQHQQQLPQNQNHYQPHYQQQQQYHQPQQHHNNQLHHQSSLTNVATHNPTATALATTTTPNTLLSTSSTSIQHISPHPYQQPPQHYHPHNYQQQSQHQSQHLHHGEFPLPEGWDIAKDFDGKIYYIDHNTKKTTWLDPRDQYTKPQSFEDCVGDELPMGWEEAYDPNIGRYYINHIAQTTQLEDPRQEWKSVQEQMLRDYLLAAQDQLENKREMYDVKQQRLLIAQEEYNHLNKLAASRSSLCSSSSSMSRHDPELLRADLKLAKERVHQLKQELKHITNDISYTERGMNTLYSVGEKINARQNGCYDIAEVQAIREEMLKVHKSLVSGEKVREELIRSLVQIKNELSRQQINEENAELMNASSPFDRVCVASQTDLCGPGDNLNSGARFAEMAKTKLQYTEWRKHIKRLQQQLADHVERIEPGQLESDKDRILLIQEKEKLLNELNSISLKSRTAEEIHVIQETRRKLEEDLNEAYEATNQQIKNRLRFHEEKQLLLDKLQEALKSTKMLEERLKSFSSESTFSISSGSSLGSLSTASSKSALSFTDIYIDPFSVDPQIDVVDLHRRSQRLFQQNPQLQQQAQILQQNQVVEQVLAHPPLQQQQSSEISLSPRSSLSMETPPASPMKYNPASEQNPLSNSSQQLLKEEPTYANAGAVLQTLYQQSNQIPACVGNRAAAPNPYDLDSTVLDCMMLEAKLQKLNMSAPLNLGAPLSPISEKPSLLDLPQEMLSRSSSTSNTRSVSAAVSNESVAGDSGVFEASRAHLPRKELAQVQIGLKYLKKEGVLVVSLERANNLSALVTTSTENSQVYLRAALLPNSLTSIRTKAMSDFQKPVFNDTFAVPISLSKLLTKSLHVTVVSMTGQKEEIIGTVQISMAEFNPEDSILKWYNVLSSKFMPTFEPLDMPSTSAAAAAAVAGHVNSNVTVSHNVCKEESSDESTITSSQTSTLTRNQVPPFEMQAQIAEDLRENLGLDVDDHDEDEDDDEDDDEEEIDDEELEGAIGNDFTKKMLDAYMDNIKQEYVDKETNTECAFPPEKSRSQQGQQSNDDRPVKRSQTFTPSAAVGKSRYICRLNRSDSDSAMHFGVTPHPFHRGAIERRSLRFHTKAPKTATKLNHTHIPRTSLDLELDLQAQHSKLDFLNDQIAKLQNLKDALEKARDNKDPLIAAWAIENEEFQRLVERGDPAKCPEEKQLQKLLMKTTKKIYKLRKTKVPKGCPDLVSFKEKISFFTRKGLSVPELPTDFMLPEADPIEEEGEEDLEDEDDEDNAAETAIAINTALVASSIRNKNLNENHSGGSASSRRQQATQNATNKTKVSTSTTTTTTITTEETEIVKTLENTKEQQNNNNAEDDDQKQRFDYVIDRTYGVEV>g14467.t1 MATCH TO: EcR Ecdysone receptor [Drosophila melanogaster]MPASSSSAAPSNSSMSSVNVKANANALKNSNALSNVITQVTLTGSTNSGGGAAGGGAVAAGIVASSSGGATAAGGSGDANGSLVFVPSKRARLELREEWISTPSPGSVPSTAPLSPSSASQNHMYGANMSNGYASPMSAGSYDPFSPNGKTVKLLNEDKEVLMLYSAMPNIQYPPPQIRVMIPAFPLPECLNGISSTSFKLKVSWFGTKLQEPLQSPGDSKWLFNMTLKKPQRISSMDE>g19689.t1 MATCH TO: EcR Ecdysone receptor [Drosophila melanogaster]MSNGYASPMSAGSYDPFSPNGKT>g3345.t1 MATCH TO: EcR Ecdysone receptor [Drosophila melanogaster]MNLSPMMYRLNHAQGQKLPLAAEQQQTQTTATQQQQQQQQQLLPSHILLQQQLDAAGSVNASNSISSSSSSSNTTSGNNSNILHNTTTTATTTNEQHVFLNNFTNTNATNDNETPPHHNNNTNTPQVLRQATSLLQNLQQQQHHHHHPHTHSQTSSSHHSRHLNNNTTNTSNNSQANNLVITNAAAAAALVAASAAVVATTGHVDVMNNSSSSVEENLKLLKTAIKSEPLQHDTTSTIITNNTILNSSNASNLSNVLSATTTSNKDDIISLAACSALANVMPASSSSAAPSNSSMSSVNVKANANALKNSNALSNVITQVTLTGSTNSGGGAAGGGAVAAGIVASSSGGATAASGSGDANGSLVFVPSKRARLELREEWISTPSPGSVPSTAPLSPSSASQNHMYGANMSNGYASPMSAGSYDPFSPNGKTEADKVKCVYRKILLKMIVKLLNEDKEVLMLYSAMPNLQYPPPQIRVMIPAFPLPECLNGISSTSFKLKVSWFGTKLQEPLQSPGDSKWLFNMTLKKPQRISSMDE>g4997.t1 MATCH TO: EcR Ecdysone receptor [Drosophila melanogaster]MKIIVDEDYYLTQKEFELYVGVQYQPFFTGRDDLSPSSSLNGFSTSDASDIKKIKKGPAPRLQEELCLVCGDRASGYHYNALTCEGCKGFFRRSVTKNAVYCCKFGHACEMDMYMRRKCQECRLKKCLAVGMRPECVVPENQCAQKRREKKAQKEKDKIQSSVCTTEIKKEILDLMTCEPPSHPTCPLLPEDILAKCQARNIPPLSYNQLAVIYKLIWYQDGYEQPSEEDLKRIMSSPDENESQHDVSFRHITEITILTVQLIVEFAKGLPAFTKIPQEDQITLLKACSSEVMMLRMARRYDHNSDSIFFANNRSYTRDSYKMAGMADNIEDLLHFCRQMYSMKVDNVEYALLTAIVIFSDRPGLEEAELVEAIQSYYIDTLRIYILNRHCGDPMSLVFFAKLLSILTELRTLGNQNAEMCFSLKLKNRKLPKFLEEIWDVHAIPPSVQSHIQATQAEKAAQEAQATTSGITTAISAAATSSSSINTSMATSSSSSLSPSAVTLSAASTPNGGAIDYVGTDMTMSGKIVVHVNNAQPNNKIDNSNDNNDDDDNHNNSMDVDEGVVLLESATNVIAFADHHVLE>g5000.t1 MATCH TO: EcR Ecdysone receptor [Drosophila melanogaster]MKRRWSNNGGFAALKMLEESSSEVTSSSNGLVLSSDINMSPSSLDSPVYGDQEMWLCNDSASYNNSHQHSVITSLQGCTSSLPAQTTIIPLTNNASLNGNQNYTNGSMNTNLTGGGNNAGSVPGMTSLNGLGQGIQVNNHTAHNHSHHLNSNSNHSNNSSHHTNGHMGTGGGGGGGGGGGGGNGGNMNINGPNIVTNAQQLSSLQASQNGQVIHANIGIHSIISNGLNHHHHHHMNNSNNMMHHTPRSESANSISSDYSPDYRLVFSSDYRLVFSPDYRLVFSPDYRLVYSPDYRLVYSPDYRLVYSPDYRLVYSPDYRLVYSPDYRLVYSPDYRLVFSSDYRLVFSPDYRLVFSPDYRY>g5932.t1 MATCH TO: sav salvador [Drosophila melanogaster]MNYLTILLCNRKPPTMLSRRSKDKQTAHKEGVVGKYVKKETPPEIPVINVWSFDDQRTKQKSKTLQRCASASPSCDGVGAVGTGARGRSGSISRNTYTDTQPDYYHARRAKSQLPPTANSMRSTLPQPPQSTQPTRRMQQYQQQQQHHPPPINSHSFYSTNNLDMPSAHSHNNNLSSSSSNYVNIEQIDRMRRQQSSPLQHSQSSYFQRSYSSNQKHGNSFDGHSNIMRERLDSMQSNMLQLPRGATPRPQTPPHHYQQQQQQQQHQLQQQQHHQHPPLTQSYSSESSPIYENPYRVSNTLSPPSLTMAESQISLSTHGNRSESPIYSNTTSMASNINHTHHQQYQHYQPQHQIHQQQSQAVHHNYNNNSNTNQMMSSYDLSSRTTPQSHNQGNTSSSMQSLYQNTNSNNMSNNSNNNSNNNHTSSSASTSTTNNNTQLHKHPSQQSLEEELPLPPGWATHYTLHGRKYYVDHNAHTTHWSHPLEREGLPVGWRTVVSKVHGTYYENQYTGQCQRQHPCLTSYYVYTTSAEPPKAIRPETYTPPTHTHNALVPANPYLLEEIPKWLAVYSEADSSKDHMLQFNMFSLQELECFDGMLVRLFKQELGTIVGFYERYRRALILEKNRRAEQERYLQELHAATVANNNLNPNAGVVNHISQQQL>g10017.t1 MATCH TO: Jafrac2 Thioredoxin peroxidase 2 [Drosophila melanogaster]MLKVHLTLLLMVLVALGSQALATVDEADSCYSFAGGSVYPAEAPKGDHTLQTTKAVISKPAPHFEGTAVVNKEIVKLSLSQFAGKYVVLLFYPLDFTFVCPTEIIAFSDRLKEFHEINTEVIACSVDSHFTHLAWINTPRKEGGLGNVKIPLLSDLTHKISKDYGVYLDDLGHTLRGLFIIDQRGVLRQITMNDLPVGRSVDETIRLVQAFQYTDTHGEVCPAGWKPGADTIVPNPKEKSKYFEKNN>g16944.t1 MATCH TO: CycE Cyclin E [Drosophila melanogaster]MDFYKSRNSNSTSSQHSNSNSSFKSHASSSNNGASYNLEYCTSSSSLSPFGTRTNLSSIASCPSSSAKTIVNVGLEEVILSISNSSSASHISASSSCSSSLALGKLTKNMCSSSRKSTTAAAAAAASSSSASSTAAATVTAAIVATSAGNKRKRCASTSFDDRDPELGFEPSAKRQQRLPSLYQSDNGSVVSSVYPSPVDEILAQEIDTRSSSECLSTGTDFEVVNDAIPDSPNSLRPDDEDLIEDDIEHEEDLDEVNDEVEHEEDVDDSDHVTDDDDVDDIVSGTASPCSTASTKITQIYNPVVAANAADVVDNASVHHGERTPGQRLSTNEKTQSSNKTSASHLNQQSKVKSFDEYLNTHYDDCMTPAADPEPPRQCPLPALSWANAHDVWQLMCKKDEQASCLRDCNMLDHHPGLQPRMRAILLDWLIEVCEVYKLHRETYYLAVDYLDRYLSAQKNVQKTHLQLIGITCLFVAAKVEEIYPPKIGEFAYVTDGACQESDILQHEILLLQALEWSISPVTPIGWLGVYMQLNVNNRTPASFQTSQKLRNNNKNTKTTSATLNIDDAFIYPQFSGLEFVQTAQLLDLCSLDVGVGNFPYSVIAAAAISHTFDRETALRCSGLDWQTIQPCARWMQPFFEVISEESHNLHLLEQNEQITTKFGLGHICPNIVTDDSHIIQTHTTTMTMFDRACLLQEQLLAMATKIKQEASPATGLMCPDGLLTPPASSRKPMGIIDEDDENHQNLKDTN>g13631.t1 MATCH TO: aos argos [Drosophila melanogaster]MLITTLYMLAIHLINVCHSTRLPLEVYELTPSRGQHNNNNININNINIGINSPAGIISSSISSNLKHKNLEYSTINSNVDSNQPPEEEEEIDLKILNSKDHEQRNQLTNPLDRRRSRQMLDMLKSSHHDLTENFNQLHSLQITAANGAGSGEAINGVSNSVHRNGKDVRILYQVGDSEDDLPICAPNAVCSKIDLYETPWIERQCRCPTINRAPEIIIHHHHKESHSHNNHNSEKYHSYYEHSKMIHQNKHLLLDAATFGGNDKKYDNLHLKKLMHKLGAVYEDDINLPSDYVLHEDNSALSSNEENVGSLLYGGEDITDNEFPSHMAMKRQQQQHLYSNAAHMRHSGHMGHQEKITYIGGCPSGLGVEDGHTIADKTRHYKLCQPVHRLPVCRHFRDYTWTLTTSAEMNSTEQIVHCRCPKNSVTYLTKREPLEDGNSGYKYLFACSPLTRLRCQRKQPCKLFTVRKRQEFIDEVNINALCQCPKGHHCPSHHTQSGVIAGETFLEDNIQTYSGYCMAND>g2922.t1 MATCH TO: Dark Death-associated APAF1-related killer [Drosophila melanogaster]MYVHTRSSSESKATALSVLISIFLLDFLKDFDIEDVLDDINLLFTRTSLDYIIKQENKDEQIFHLIWILQTMHDDQIREFLKKIEKYYDWIVRKIENEVKCHSDETIKYLIKLSSMQSKNHKHTKYNVHRTKEYIALRSALRKLKPHNSVVLLGGLSSGKKWLAYDVCSDFDVLKSMKFKIFWIDCSECISQEKDYEALKNLMYQLKHDHQFYNVNYTKENFIRLKEQIKCLFSEKENNECLLVLCNVQNNKCINAFNLECKRLIITRNRKVSDSMPTSLNKHLELNKGLTLNEFYLLIDKYKNANYDWRMDSIPLANDIYHMSLGEPCSLSIIAKHISQKKSNWIECMKNINNFEISNRKLKQEIEKSLEIFTPDELKLFATLSVFPRCAQIPVKLLAFLWNMRTYETEHFINKLHDHFFVQRVLLDDQETITCCVKFMYSSYIRNCNNIKSIIDPLELHSRIVDFYEVKDHLNNRKDVDIDIYSYDGYFFHCIGFHLYRSSYKHLLPTLYTDFGFLGQKIRLVGLNDTIADLHTYSVEIASAERNFSLRKFENFLAKIEEKIKYFPDCCLLQYALFSTEDFIKELALKQVKKFPQRLWFTERNQYQQWRPIFNLPETAKLIRLLDDDLCIVVLSNHKVLLIDLTLGSKCCEMPLMKGINIVDMKIIDDYNHVLTQDCYGSLKLWNITEEKRCISKQRSNANFKPRLKDDTNKKFFHEQIISNYYNPSEKIQTFYVDPNNNRILVAFGGGLLMFFDWNKKDKKYKKSFENPYNVKIKTIHSVCLLNEKHYMIIYYKENNKIDVQFLKRKNTQLENLELKFPDNDKLIYHEVFQNYVIFVFQRYILRLNLIFDSFEKSNLELLYENHDNIINCAKLLLDNQYLILGTQKGINVFDITKRSVTLQSIVSESISSIDNYDLDDDKYKSMIVCGCNHKNILYIFGLHLTNGSKLDWDHNPLRQNESDELASFEQIYTRYIGNKLFDVCEENDTLYAVDSKNRIHQICLSDTKKISILPSLDVQQNIIGIHCFKSKLIVAFNNGEIYDYLEKRLLVKANDVVEKDFFLKTMDNNILITSNANELTYFKHMQIEHSEKLLQTQTKYCKIIMNTHLLIINNCGAALIFDCEGFFKNPDKVNIVIGIKACNNLSSCDLKDNVLFLGFSQYAVHIYTLDKDSNGSISCLKRSEIPTVNSLRITCLAASNNKLLAIGLQNTTSEDLRSTGDIEIWKLIDDYKPQLMFCLKSHVKPINDLRFSPHNDVLISVAEQICFWNMNYVINNPLDLGNKKRHSSRFNSQKSMEEVDFKVNSKHQRLGGDNLLNLNYMLINSPEFKNNVLVLNNSLEQTITSDELLENNVFSASVEKDDGSNWSGLTGSSDKPELLSCLKLDGNEAQQIFIHKNFTQFSTIDDEGVYYSLERFTMSSPEDTAEDEDKTDSSSSSVNLHPDVHRFSIDLSSNMPGTDVVDTANS>g13486.t1 MATCH TO: Cul3 Cullin 3 [Drosophila melanogaster]MNIRSTAPKKEGKMRIRAFPASMDEKYVETIWASLKNAIQEIQKKNNSGLSFEQLYRNAYNMVLHKYGHRLYFGLREVVKEHLEEKVRKDVLESLHNNFLPKLNQAWTDHQTSMVMIRDILMYMDRVYVQNREVENVYNLGLILFRDEIVRYSEIQKALRETLLGMVMDERSGEAINHLAIKNACQMLMALGINSRSVYEDDFEKPFLAQSASFYKFESQKFLAENNASVYLKKVETRIQEESARAALYLDKDTEPRIVKVVEDELIKKHMRTIVEMENSGVVYMIKNSKTEDLACTYKLFSRLKDEGLKVIADTMSAYLREQGRMLVKEEENGTTNPITFVQNLLDLKDRFDQFLHHSFNNDRLFKNVISSDFEHFLNLNSKSPEYLSLFIDDKLKKGGKGMTEQEIEVILDKTMVLFRFLLEKDVFERYYKTHLAKRLLLNKSVSDDFEKNMISKLKTECGCQFTSKLEGMFKDMSISNTTNEEFKIYVNNHNINLSVEFTCRILTTGFWPTQTSTPNCNIPAAPREAYEIFKKFYLDKHSGRQLTLQPQLGNACLNAVFYGRKVVDAAAAAAEKDKDAPSSSMSSSSSSGCTIPATTRKHILTVSTYQMCILMLFNNREVMTYEEILQETDIPERELQRAIQSLSMGKPSQRLLVRNSKTKTKDIDPSDEFLVNDAFVSKFHRVKIQTVAAKADTEPERKETRGKVDEDRKHEIEAAIVRIMKARKRMPHNLLVSDVTSQLKSRFLPSPVLIKKRIEGLIEREYLARTPEDRKVYIYLA>g15874.t1 MATCH TO: Dp DP transcription factor [Drosophila melanogaster]MAHSSSATAASATTNEINYFFKDENGKIHKVLKQVEIKQEPLGGSSTVYTSVGGNVSTFTQMNTNQPQFVRIQNNYNIQTTENPTYTVITPQSSSGSSSSNMQLRQQQQQQHTNNTNITTIKYEPVEEFTTHTAVVKEEERYKYKPQILTNTAKTTTSKSKIQQQSTSHQNVSSAASVAAVQRKRKPEKAGKGLRHFSLKVCEKVKVKGTTTYNEVADELVAEEIQNNSFDKNCDQKNIRRRVYDALNVLMALQIISKDKKEIRWIGLPSNAVEEFSQLEKDNQERRLRIKQKNQQLRELLLQQVSFKSLIERNKEAERQGIIPTPNSSIQLPFIIVNTHKSTKINCSVTNDKSEYIFKFDDKFEMHDDAEVLKRMGLLFGLEKGQCSDEDIERAKSLVPPNFEKYIEAYGSGKASDISYDSDDEMNSYDYIELAQDNSQQSFTTSTTTTHNALVSSGGGDGGGGGVGVIIKSNDLDDDEEYVEHSDID>g20009.t1 MATCH TO: Clbn Caliban [Drosophila melanogaster]MKLRFNSFDIICGVTELQSLVGLRVNQIYDIDNKTYLIRLQGGGEAKKVLLIESGIRFHTTAFEWPKNMAPSGFSMKLRKHLKNKRLEKVQQLGFDRIVDFQFGIGEAAYHIIVELYDRGNIVLTDHQMAILYILRPHREGEDVRFAVREKYPIERAKKVSPLPSEDELRNIIFHTAEGETLKHIIMSVIDCGPSVIEHALKKHDLDKCIIPKVDISEKVNEGREKVIKDLNKKTKKNKRNNRNQLNTCDFNIETDINSLMLVLQDISEIIQGAQRNISKGYIIQKKEEKPSTEERKDIEHFYQNVEYHPYKFCQYEGEPIVEFETFMLAVDEFYSTLEGQKIDMKTLQQERDALKKLSNVKKDHAKRIEDLNRVQEGDKQKAELITVNQGLVDNAILAIRSAIANQMSWPDIHELVKAAQANGDPVAGAIKQLKLEINHISLKLSDPYDTGTKDACNQQRENSNKPTIVDVDLALSAWANARKYYDQKRSAAQKEKKTIDASQKALKSAERKTQQTLKDVRTICTISKARKVYWFEKFYWFISSENYLVIGGRDAQQNELIVKRYMRPNDIYVHAEIQGASSVIVRNHEGGDIPPKTLLEAGTMAISYSVAWDAKVVTNAYWVRSEQVSKTAPTGEYLGAGSFMIRGKKNFLPSCHLIMGLSLLFKLEDSFAERHKGERKVRTFEEEQEHQAENRLKSFSLEGEHLEDIAEVELEEENEGNDSNEENKCYETMKETEKFEEQIDEEFPDTQVKVEHDIGRISLENKHTGILDYNLLTNIEEEESTIIPAPPRKKLQQNSKKRKEEKDRKSQQTQKLQHADDSQNKHNSQVVKRGQKGKLKKIKSKYKDQDDEERQLRMQILKSAGKIKEIRNDTMEDEEISFAKHFKQVKRQNDEKQLLQTDAGDFDDIPTSANVEMIDSLTGQPFEDDELLFVIPVVAPYQSLQNYKFKVKLTPGTGKRGRAAKTALTMFAKDKMCTSRERDLLKSIRDEAVARNIPGKVKLSAPQLQKFKK>g3635.t1 MATCH TO: Mdh2 Malate dehydrogenase 2 [Drosophila melanogaster]MLKQVTKQLAIQSMRSFSTSRQNNYKVTVCGASGGIGQPLSLLLKQNALVTDLALYDIVHTPGVAADLSHIDTKSKTTGYMGADQLGQALKNADVVVIPAGVPRKPGMTRDDLFNVNAGIIKDIANSIAKNCPKAMVAIITNPVNTCVPIAAEVLKKAGVYDPKRLFGVSTLDVVRARAFIGEALGVDPQKVNIPVIGGHSGVTILPVLSQCQPAFKGDQSTIEKLTVRIQEAGTEVVKAKAGAGSATLSMAYAGARFAGSLLRGLNGESNVVECSYVQSNVTEATFFATPLVLGKSGLQENLGLPKLNDFEKKLLEAAIPELKKNIQKGIDFANA>g3652.t1 MATCH TO: Mdh2 Malate dehydrogenase 2 [Drosophila melanogaster]MLKQICKTLRSSPGKRLISTSLPHLHRVTIIGSAGGIGQALSMLIKADPLVTSLTLMDIQNTAGIAADISHIDTFSDVKSFTGAEHMEESLECCELVVIAAGIARSEKMSPTDLFNANKELIHHVALAKAKICPEAFMAIITNPVNSLVPLAAETLKQANAYDGKKLFGVTKLNTMRSRAVVADYSLIDPHKVKVNVIGGNSPETIVPLLYSASPKIQGDCDELEKISERIRKSPIKGKEFAQLSTAYAAATFCNSLLKALDGDRGVTDVAFVESAVFPEVKFFSSNIELNKSGIKDFEALPTMSAYEKDLLKKAISEIQKDIDHGVQHAGSKKEKK>g7135.t1 MATCH TO: Mdh2 Malate dehydrogenase 2 [Drosophila melanogaster]MLKQVTKQLAIQSMRSFSTSRQNNYKVTVCGASGGIGQPLSLLLKQNALVTDLALYDIVHTPGVAADLSHIDTKSKTTGYMGADQLGQALKNADVVVIPAGVPRKPGMTRDDLFNVNAGIIKDIANSISKNCPKAMVAIITNPVNTCVPIAAEVLKKAGVYDPKRLFGVSTLDVVRARAFIGEALGVDPQKVNIPVIGGHSGVTILPVLSQCQPAFKGDQSTIEKLTVRIQEAGTEVVKAKAGAGSATLSMAYAGARFAGSLLRGLNGESNVVECSYVQSNVTEATFFATPLVLGKSGLQENLGLPKLNDFEKKLLEAAIPELKKNIQKGIDFANA>g8612.t1 MATCH TO: Mdh2 Malate dehydrogenase 2 [Drosophila melanogaster]MLKQICKTLRSSPGKRLISTSLPHLHRVTIIGSAGGIGQALSMLIKADPLVTSLTLMDIQNTAGIAADISHIDTFSDVKSFTGAEHMEESLECCELVVIAAGIARSEKMSPTDLFNANKELIHHVALAKAKICPEAFMAIITNPVNSLVPLAAETLKQANAYDGKKLFGVTKLNTMRSRAVVADYSLIDPHKPHLKFKGDCDELEKISERIRKSPIKGKEFAQLSTAYAAATFFCGNSLLKALDGDRGVTDVAFVESAVFSGSKFFSSKYRIE>g8613.t1 MATCH TO: Mdh2 Malate dehydrogenase 2 [Drosophila melanogaster]MFDANAELMDVGSYDPRKIIGSSSVDAMRSRTFLGQVVNFDPSKIFVPVIGGHSAGSITPLVSQSKPDFELDANVQKKLEDRIVHGASEVVKAKNAKGAAQLAMGSLCGENFVIQCVVSFMVKEMWWSLDNDGIKECHRLPPMSEYETELYCKAVKDVQSNINNAKQFSKNINRRRVNENFKDV>g18961.t1 MATCH TO: Mdh2 Malate dehydrogenase 2 [Drosophila melanogaster]MLKQICKTLRTSPSKRLISTSLPHLHRVTIIGSAGGIGQALSMLIKADPLVTSLTLMDIQNTAGIAADISHIDTFSDVKSFTGAEHMEESLECCELVVIAAGIARSEKISPKDLFNANKELIHHVALAKAKICPEAFMAIITNPVNSLVPLAAETLKQANAYDGKKLFGVTKLNTMRSRAVVADYSLIDPHKVKVNVIGGNSPKTIVPLLYSASPKIQGDCDELEKISERIRRSPIKGKEFAQLSTAYAAATFCNSLLKALDGDRGVTDVAFVESAVFPEVKFFSSNIELNKSGIKDFEALPTMSAFEKDLLKKAISEIQKDIDHGVQHAGSKKEKK>g18962.t1 MATCH TO: Mdh2 Malate dehydrogenase 2 [Drosophila melanogaster]MWNSLRRMSQQTKVVEMCKICIVGANGGIGRHLALNLKLNKYVTDLSLYDIVETASLREDLLNIPTRTTITNHTGDEKALIESLTCSNIVVILAGQAHSPKFTSREAMFDANAELMVKFMKATVAACKNRMPFIHIVTNPVNALVPLCAEYLKDVGSYDPRKIIGSSSVDAMRSRTFLGQVVNFDPSKIFVPVIGGHSAGSITPLVSQSKPDFELDANVQKKLEDRIVHGASEVVKAKNAKGAAQLAMGHCVAKFCDSVCRVIYGERNVVEVGYVPADVNELEFFACNFSLDNDGIKECHRLPPMSEYETELYCKAVKDVQSNIDNAKKFFQKYKSKKSK>g4215.t1 MATCH TO: hppy happyhour [Drosophila melanogaster]AKRIQSNELAAIKVIKLEPTDDIQIIQQEIIMMRDCRHPNIIAYYGSYLRRDKLWICMEYCGGGSLQDIYQVTGPLTEQQIAYMCRETLKGLEYLHSMGKMHRDIKGANILLTEYGDVKLADFGVSAQITATINKRKSFIGK>g6226.t1 MATCH TO: hppy happyhour [Drosophila melanogaster]MAHHNANLLSSDISRRNPQDEYELIQKIGSGTYGDVYKVSLLNFKYRIFGPQVGTTDAADGDDDDDLVVFYIEFICGCKVHHFVQDEKK>g11426.t1 MATCH TO: hppy happyhour [Drosophila melanogaster]MAHHNANLLSSDISRRNPQDEYELIQKIGSGTYGDVYKEEKCVNSPFSHSTTSSCQASSKSNDWNIGAK>g14955.t1 MATCH TO: hppy happyhour [Drosophila melanogaster]MYLMDESLIYRTGFEQKAKRIQSNELAAIKVIKLEPTDDIQIIQQEIIMMRDCRHPNIIAYYGSYLRRDKLWICMEYCGGGSLQDIYQVTGPLTEQQIAYMCRETLKGLEYLHSMGKMHRDIKGANILLTEYGDVKLADFGVSAQITATINKRKSFIGTPYWMAPEVAAVERKGGYNQLCDIWACGITAIELAELQPPMFDLHPMRALFLMSKSGFKPPTLNNKDKWSPTFHNFVKTALTKNPKKRPTAERLLQHPFVMGEMSVRVAKELLKKYMNPNQQFYYNWDGDEETVASVPQRIASKMTSRPNGISPQNHTLKTGMTSSSSQWNNERSSSPETLPSDMSLLQYIDEELKLRATLPLTDTKDLLSTECNCSHNGGGNSNHNNTSSSSATSVVASAATIVTTATSTSSSLSLNNGCATDVTATATTNSSLLGGSSCSSGVHTTTNGGNSSLATSVAYGVGGIGGNNGLSNIRTTAVDHVDNHSSLFAHFDMLRHGGHHNNYNNTNYNRPISTPASGLITNANLLSGGSSGGATTNGQMSTSTSMYSHHLLDNSSSDLRGGGGVDIYHGGGGGGGVIGSSSNIISSSGIGGHHHGSSVGSGGGAGGGGGVGGSHYHHHHHHHHRHHSQDSYTSASPSSVSALSSYLHINNSSGSSSVVGGGGGVGIGGHLHSSSSSSSILYSSNANTSSSASTNACHTSNINSNSISYIPSTCPSPSASSSTYTNNHNNHNVMSTVMSASICSNSASPPLSSYNNSTTTSHFYNNLLRNNGSNYLNSNTTSDVVASTSANSSSNFSSASALTNNPQMSTNAETTTTTNNIANNSHHNCDYRHENNQNGLDDSPRRHSSMDQLLGLLNEMGKSSRTRSLSDGGTQEDDDELDKEAQPDLLNNTPPVPPKRSHRRRHTPPRPISNGLPPTPKVHMGACFSKIFNGCPLRVHCTASWIHPETRDQHLLIGAEEGIFNLNMNELHDAAIDQLFPRRTTWLYVIKDVLMSLSGKSCQLYRHDLIALHSKQTHRFSLHMNKIPERLVPRKFALTTKVPDTKGCTQCCVTRNPYNGYKYLCGATPNGIFLMQWYDPLNKFMLLKQCEWPATSILGGGHGCVQNGHTPVFEMIITPELEYPIVCTGVRKAANGCLKLELINMNSASWFHSDDLEYDAMATMVPRRDLLKVVKVHQVEKDAIIVCYGNIIQVVTLQGNPKQHKKLVSQLNFDFNVDSIVCLPDSVLAFHKHGMQGKSLRNGEVTQEIKDMSRTYRLLGSDKVVALESQLLRTGSLGSEEGHDLYILAGHEASY>g14955.t2 MATCH TO: hppy happyhour [Drosophila melanogaster]MYLMDESLIYRTGFEQKAKRIQSNELAAIKVIKLEPTDDIQIIQQEIIMMRDCRHPNIIAYYGSYLRRDKLWICMEYCGGGSLQDIYQVTGPLTEQQIAYMCRETLKGLEYLHSMGKMHRDIKGANILLTEYGDVKLADFGVSAQITATINKRKSFIGTPYWMAPEVAAVERKGGYNQLCDIWACGITAIELAELQPPMFDLHPMRALFLMSKSGFKPPTLNNKDKWSPTFHNFVKTALTKNPKKRPTAERLLQHPFVMGEMSVRVAKELLKKYMNPNQQFYYNWDGDEETVASVPQRIASKMTSRPNGISPQNHTLKTGMTSSSSQWNNERSSSPETLPSDMSLLQYIDEELKLRATLPLTDTKDLLSTECNCSHNGGGNSNHNNTSSSSATSVVASAATIVTTATSTSSSLSLNNGCATDVTATATTNSSLLGGSSCSSGVHTTTNGGNSSLATSVAYGVGGIGGNNGLSNIRTTAVDHVDNHSSLFAHFDMLRHGGHHNNYNNTNYNRPISTPASGLITNANLLSGGSSGGATTNGQMSTSTSMYSHHLLDNSSSDLRGGGGVDIYHGGGGGGGVIGSSSNIISSSGIGGHHHGSSVGSGGGAGGGGGVGGSHYHHHHHHHHRHHSQDSYTSASPSSVSALSSYLHINNSSGSSSVVGGGGGVGIGGHLHSSSSSSSILYSSNANTSSSASTNACHTSNINSNSISYIPSTCPSPSASSSTYTNNHNNHNVMSTVMSASICSNSASPPLSSYNNSTTTSHFYNNLLRNNGSNYLNSNTTSDVVASTSANSSSNFSSASALTNNPQMSTNAETTTTTNNIANNSHHNCDYRHENNQNGLDDSPRRHSSMDQLLGLLNEMGKSSRTRSLSDGGTQEDDDELDKEAQPDLLNNTPPVPPKRSHRRRHTPPRPISNGLPPTPKVHMGACFSKIFNGCPLRVHCTASWIHPETRDQHLLIGAEEGIFNLNMNELHDAAIDQLFPRRTTWLYVIKDVLMSLSGKSCQLYRHDLIALHSKQTHRFSLHMNKIPERLVPRKFALTTKVPDTKGCTQCCVTRNPYNGYKYLCGATPNGIFLMQWYDPLNKFMLLKQCEWPATSILGGGHGCVQNGHTPVFEMIITPELEYPIVCTGVRKAANGCLKLELINMNSGASWFHSDDLEYDAMATMVPRRDLLKVVKVHQVEKDAIIVCYGNIIQVVTLQGNPKQHKKLVSQLNFDFNVDSIVCLPDSVLAFHKHGMQGKSLRNGEVTQEIKDMSRTYRLLGSDKVVALESQLLRTGSLGSEEGHDLYILAGHEASY>g18321.t1 MATCH TO: E2f1 E2F transcription factor 1 [Drosophila melanogaster]MSKYFINTTTTNANAAATSNVYHVSQNVGTTTSSTTNSSVVSTTGTSSAIRKLFSENSSGTKTHNTATSTTRGSSNAKATRIFMTTNQAQQQHHQQVQQNASNTTTTHMATHLLDHGYGVTLTPPTVSTSVVVGQETTSVLSTTSNSAPTSGTVSVTSNTIPASQQQQQQQHPQKFKPTDMTQYYKVKRRIPMNDSHPKKQAKQSLQHQTPYQQQQQQHVVIRSNTMKQQTPQIHHQHQHQQQQQQQLSLNFATPSPQPPRPPSSASSTSSSSALMAPNLFYKIGTASGNNHGNNNTGSSSKRTPEGNRADTSLGILTKKFVDLLQESPDGVVDLNDASAKLAVQKRRIYDITNVLEGIGILEKKSKNNIQWKCGNSLLTSERSNDIKLENERLEQKENELNMLIDEIRNELHGEISRNDQLAYVTHSDLLNVDLFKDQIIIVIKAPPEAKLVLPDSLNPREIHVKAENNGEINVYLCHDNSPENSPNFGSSSLAQRHANAAIQQRQHDPLITDMDAEKRHRLGLTGVSRQPLSSTYPASATNLVQRSAQRNLSKSIEAAAQQEAAASLLEVNYTTQDQQQMEHHHHLQQHEQSSSSLLNEEEFNMFPSITRPVITQVLTDQHHRLQNSQEQKLSVEEDALASYQNLIACSNQQQRRSTGESNPDSNDSNQTLTLNSCSNTTNSSNLLSKKSGLRNDVTLVNCAMEPQLQQTSLLKGGATSSSSSSQVPSNNSSSATSPQHQQQDNSPTHLSYETLTHNNNQQMQTTTAGGHQQQQLQHHQQHQHHQQHHHHHQQQQHQDLQQQLNNSIVVNSSTNNDDLSQMHVSNSNCNTGVRNALISDSVNLSPTSYNYFEDLPPLLPIEPPLEGLYNFSLDQSEGLNDLFHDFV>g13214.t1 MATCH TO: rdx roadkill [Drosophila melanogaster]MSSPTSPFSSPSSSSSSSEYSPSTSSPESNSSSSYLNSIKVEFDRNIMELVYQPRLPVNECQASQTARVTSNLNASSSTMAVSRVPSPPLPEVNTPVAENWCYTQVKVVKFSYMWTINNFSFCREEMGEVLKSSTFSAGANDKLKWCLRVNPKGLDEESKDYLSLYLLLVSCNKSEVRAKFKFSILNAKREETKAMESQRAYRFVQGKDWGFKKFIRRDFLLDEANGLLPEDKLTIFCEVSVVADSVNISGQSNIVQFKVPECKLSEDLGNLFDNEKFSDVTLAVGGREFQAHKAILAARSDVFAAMFEHEMEERKLNRVAITDVDHEVLKEMLRFIYTGKAPNLDKMADDLLAAADKYALEKLKVMCEEALCVNLSVETAAETLILADLHSADQLKAQTIDFINTHATDVMETQGWQNMITTHSHLIAEAFRALATQQIPPIGPPRKRVKMS>g13215.t1 MATCH TO: rdx roadkill [Drosophila melanogaster]MFDPFVKIKKKQNIFNDLEPIEIVDDINENSSDTDNDCCCLTPPPTAASTPLLNITPSTSSTSSSSSTSTSSSSSSSSATNTSSTSSSSSSLAVANASELLNTSSASSTTTTTTAASTSSLNLQNCSRLQQLIAAPPIIQTTHSQQQQQQQQQLNTPPTPPPSTPPTTPALHRSDILQQHLQASTTGSKQLSGQQISVLQQHLGHLPLTSTATTERSSSTSNTTLHNLARHLSTPSSLYNSSSSSSTSSSSGSSSPALLQEVFSTNNLQNVLKRQSSSISNTSTNSNSTTSSSSSSSSSSSSSLSSPANSTLRSLVSNPSSLITSSTTLNSIIANRLNSPPALAQNSHITAGTSTASTSSSSSSSSSSSAAQSLYHQQHSALTNSITHRIHQSIRRHLNQQQNQVLATTQQQQSSGQQHQSNHQQQSRIQNLQQNSQLQNHYHHHHHHQHQPQQHSSSSSSSSSSSLSNSSGSSSSSSSSSSSSSSANNNNSPLCVVLLVKCPNSKEYCNALQQQQQQQQQNNQNLNQLINPNPATLNLPQQHQQHHHHHHHHHQQQQHLHCCDNKR>g10291.t1 MATCH TO: RnrS Ribonucleoside diphosphate reductase small subunit [Drosophila melanogaster]MMSGKENIIDNMDKFSLKSPRKILTAVNVGNVRKMSIGDDLQLNGKSQDIKDLNSLDSGSQNKNGAGTSLANKAPVPFDPSIEPLLKENPRRFVIFPIQYLDIWQMYKKAEASFWTVEEVDLSKDLTDWERLTDNERHFISHVLAFFAASDGIVNENLVERFSQEVQVTEARCFYGFQIAMENVHSEMYSILIDTYIKDRDQREYLFNAIETMPAVKRKADWALSWISSKSANFGERIIAFAAVEGIFFSGSFASIFWLKKRGLMPGLTFSNELISRDEGLHCDFAVGSKIEQEFLTDALPVNLIGMNCKLMSEYIEFVADRLLVELEVGKIYNTKNPFPFMEMISLDGKTNFFERKVGEYQKLGVTAHRLDNVFTLDADF>g19516.t1 MATCH TO: lok loki [Drosophila melanogaster]MVRDVELSEQPQTQGATQSQASNLWSQVESQPMDNIVWGRLYGKNIKVKSLDLNIETFTAGRGENNDLILTLNDLPEKILCRISKVHFTITRAGCDLSNPVYIEDKSRNGTFVNSERIGTNRRRILQNDDIISLSHPTYKAFVFKDLSPNEAMGLPIDITSNYYISRKLGSGACGLVRLVYDRRTCQQYAMKIVKKNMLATSTNPNHLNDPNRVMNEAKIMKSLEHPCVIKMHDIVDKPDSVYMVLEFMKGGDLLNRIVSNKRLSEKVSKLYFYQMCHAVKYLHDKGITHRDLKPDNVLLETSNEETLLKVSDFGLSKFVQKDSVMRTLCGTPLYVAPEVLLTGGRGAYTKKVDIWSLGVVLFTCLSGTLPFSDDYGSPACDQIKKGQFRFNHPSWKLVSQRATSLIKNMLNVDPQRRPTIDDVLQSSWLKDPTMLRTANKLMNIEPMETEDQENFIEPPTKRSRR>g13942.t1 MATCH TO: Sep1 Septin 1 [Drosophila melanogaster]MSETKSFSSIETPGYVGFANLPNQVHRKSVKKGFEFTLMVVGESGLGKSTLVNSLFLTDLYPERVIPDAIEKQKQTVKLEASTVEIEERGVKLRLTVVDTPGFGDAIDNGDSFSAILEYIDEQYERFLRDESGLNRRNIVDNRIHCCFYFISPFGHGLKPLDVEFMKKLHSKVNIVPVIAKADCLTKKEILRLKCRIMQEIEDHGIKIYPLPDCDSDEDEDYKEQVKQLKAAVPFAVCGANTLLEVKGKKVRGRLYPWGVVEVENPDHCDFIKLRTMLITHMQDLQEVTQEVHYENYRSDRLAKGIKNKENGVKPERDSLVPTQIVVNSVLSEKDRILQEKEAELRRMQEMLAQMQAKMQAQQ>g18486.t1 MATCH TO: Sep1 Septin 1 [Drosophila melanogaster]FSSIETPGYVGFANLPNQVHRKSVKKGFEFTLMVVGESGLGKSTLVNSLFLTDLYPERVIPDAIEKQKQTVKLEASTVEIEERGVKLRLTVVDTPGFGDAIDNGDSFSAILEYIDEQYERFLRDESGLNRRNIVDNRIHCCFYFISPFGHGLKPLDVEFMKKLHSKVNIVPVIAKADCLTKKEILRLKCRIMQEIEDHGIKIYPLPDCDSDEDEDYKEQVKQLKAAVPFAVCGANTLLEVKGKKVRGRLYPWGVVEVENPDHCDFIKLRTMLITHMQDLQEVTQEVHYENYRSDRLAKGIKNKENGVKPERDSLVPTQIVVNSVLSEKDRILQEKEAELRRMQEMLAQMQAKMQAQQ>g16066.t1 MATCH TO: Atg13 Autophagy-related 13 [Drosophila melanogaster]MSTQRINAAEKDLEKFIKFLALKSTQVVVQSRLGEKIQTKCNPMAGNDWFNLVVEDHPDVYAETKKALGLSPGESILKRLPLCVEISLKTAEGDQMILEVWSLDLQPSNSQKSKETQEKGQNQNINSTASEQPCLKTAHAIYNRMGMLLKSLISLTRATPAYKLSRRQCPDSYSIYYRIYVERPQVHTLGEGHKNVRIGHLNTIVGNLVMSVAYRTKMTITPTATTGQTRTRESNAIMIDSNHFKHCDTGCKQNGSGLNGKKSAGQGGEKKIIDIEKPLRPGAFTDIGKLRQYTEDDFVLPETPPFEWLLRKPRHESAGSAGTGCGGSIESLNRKCDSPQTSPLGNGHQNTININNALNNNSSNGNSNNANNTTTNNNSAAFKSLENDENLQQLQLQQQQQQKSSPNNSTHSQSPIKSLFVPQPPLNARLQPHNSTPNLPATTQHSADDESLLKELNFPFASPTSHVNDLAKFYRDCYHAPPLKGFSELQAELCSTINNTTTAAYTSCNTQNTPAIDNPLTCSATAANNTSSTATTTNTTDYTFVDDLSKQLEQFETSLEDYDKLVSQFGILQTSSSTGSRSSGGLQMSN>g20884.t1 MATCH TO: RpS3 Ribosomal protein S3 [Drosophila melanogaster]MSLTISKKRKFVADGMFKAELNEFLTRELAEDGYSGVEVRVTPARTEIIIMATKTQQVLGEKGRRIRELTAMVQKRFNFEPGRIELYAEKVATRGLCAIAQAESLRYKLTGGLAVRRACYGVLRFIMESGAKGCEVVVSGKLRGQRAKSMKFVDGLMIHSGDPCNDYVETATRHVLLRQGVLGIKVKIMLPYDPKNKIGPKKPLPDNVSVVEPKEEKIYETPETEYKMLAVVPIYDEAADM>g4778.t1 MATCH TO: CG5059  [Drosophila melanogaster]MSATPPRMNEDLLGESWIELSTAATMAGVKSPDRITPLPFANGEEYLRLLREAQRESNQSSRVVSLASSRRDTPRDSPKSPPNSPNTELCAEDELKNVYINYWNKTGETSKDTDWLDEWNSRPDQQPPKDWKFEHPQNQQKKKTAGYSIRLTRVGKNSLFSREILYSLILSNVLSLLLGAGLGLWLSKRGILFTRVVID>g18237.t1 MATCH TO: p53 p53 [Drosophila melanogaster]MFLIYERSIMTSQRHQSVLQQLIQEQIDIKPNLNLIPTCEEHDHGGYNFRIQINCSDKYSQRPGVVYSEETQRLYLKTNENVYIDCFYTQRMPIQPLKVRVFAIFEKDASDPVLRCQNHISTDNDPDETVRKSLVRCKNPDAVYYGSDTGKSINDRYSVVVPLNSTVKGSETNQLKQQLIVSFTCYNSCMNRKQTAVIFLLEGMNGEILAQRTLSVKISTCPKRDRRLYESAEINLNKRKHIEEPATEAKMAKYENTSIKDEYSRSSFDETSDDGISSTFSGSVHHEKDTGDYVLTLKYKSRQHILQAIKAIYADTIANEELCRTSINGRRTRDSDYIKKLYTTAYNIKYS
