# Supplementary material for: Genomic analyses of a livestock pest, the New World screwworm, find potential targets for genetic control programs
Source: Commun Biol. 2020 Aug 4;3:424. doi: 10.1038/s42003-020-01152-4 (PMC7403345; doi:10.1038/s42003-020-01152-4)
Supplement: Supplementary file 14 — Reporting Summary [file 42003_2020_1152_MOESM14_ESM.pdf]

nature research

Corresponding author(s): Maxwell J Scott

Last updated by author(s): Apr 27, 2020

Reporting Summary

Nature Research wishes to improve the reproducibility of the work that we publish. This form provides structure for consistency and transparency in reporting. For further information on Nature Research policies, see Authors & Referees and the Editorial Policy Checklist.

Statistics

For all statistical analyses, confirm that the following items are present in the figure legend, table legend, main text, or Methods section.

n/a	Confirmed	
☐	☒	The exact sample size (*n*) for each experimental group/condition, given as a discrete number and unit of measurement
☐	☒	A statement on whether measurements were taken from distinct samples or whether the same sample was measured repeatedly
☐	☒	The statistical test(s) used AND whether they are one- or two-sided<br>*Only common tests should be described solely by name; describe more complex techniques in the Methods section.*
☐	☒	A description of all covariates tested
☐	☒	A description of any assumptions or corrections, such as tests of normality and adjustment for multiple comparisons
☐	☒	A full description of the statistical parameters including central tendency (e.g. means) or other basic estimates (e.g. regression coefficient) AND variation (e.g. standard deviation) or associated estimates of uncertainty (e.g. confidence intervals)
☐	☒	For null hypothesis testing, the test statistic (e.g. *F*, *t*, *r*) with confidence intervals, effect sizes, degrees of freedom and *P* value noted<br>*Give P values as exact values whenever suitable.*
☒	☐	For Bayesian analysis, information on the choice of priors and Markov chain Monte Carlo settings
☐	☒	For hierarchical and complex designs, identification of the appropriate level for tests and full reporting of outcomes
☐	☒	Estimates of effect sizes (e.g. Cohen's *d*, Pearson's *r*), indicating how they were calculated

Our web collection on statistics for biologists contains articles on many of the points above.

Software and code

Policy information about availability of computer code

Data collection	*Provide a description of all commercial, open source and custom code used to collect the data in this study, specifying the version used OR state that no software was used.*
Data analysis	Data analysis for the RNA-seq studies were conducted with CLC Genomics Workbench (available through Qiagen), EdgeR (published) followed by WGCNA (published). Software for the prediction of transcription factors and their binding sites has been published. All other software used is open source. Trimmomatic version 0.23, Canu version v1.4, Arrow version 2.1.0, Tophat version 2.1.1, Braker v1.9, GeneMark-ET (v4.33), Augustus (v3.2.3), RepeatModeler2, RepeatMasker v. 4.0.7, MAFFT, GBlocks, RAxML version 8.2.8, FigTree version 1.4.2, OrthoFinder (v 2.2.7), MUSCLE, Geneious v6.1.8, g:Profiler, REVIGO

For manuscripts utilizing custom algorithms or software that are central to the research but not yet described in published literature, software must be made available to editors/reviewers. We strongly encourage code deposition in a community repository (e.g. GitHub). See the Nature Research guidelines for submitting code & software for further information.

Data

Policy information about availability of data

All manuscripts must include a data availability statement. This statement should provide the following information, where applicable:

- Accession codes, unique identifiers, or web links for publicly available datasets
- A list of figures that have associated raw data
- A description of any restrictions on data availability

The genbank accession for the genome assembly is ASM430292v1. The Illumina and PacBio reads from genomic DNA and the RNA sequencing data were deposited at NCBI under SRA accession PRJNA641284. The datasets supporting the conclusions of this article are included within the article and its supplementary files. The supplementary data files are available at Dryad, https://doi.org/10.5061/dryad.x69p8czg2

Field-specific reporting

Please select the one below that is the best fit for your research. If you are not sure, read the appropriate sections before making your selection.

☒ Life sciences ☐ Behavioural & social sciences ☐ Ecological, evolutionary & environmental sciences

For a reference copy of the document with all sections, see nature.com/documents/nr-reporting-summary-flat.pdf

Life sciences study design

All studies must disclose on these points even when the disclosure is negative.

Sample size	For RNAseq, RNA was prepared from three independent collections from each stage examined.
Data exclusions	No data was excluded
Replication	For RNA seq, the life stages were collected from independent cages of insects. For each stage the insects were collected within the specified time window. eg Embryos were collected 0-1h after egg laying.
Randomization	We are simply presenting a developmental RNA expression profile for genes. There was no control and experimental test.
Blinding	Blinding was not necessary as there was no control and experimental test for RNA expression.

Reporting for specific materials, systems and methods

We require information from authors about some types of materials, experimental systems and methods used in many studies. Here, indicate whether each material, system or method listed is relevant to your study. If you are not sure if a list item applies to your research, read the appropriate section before selecting a response.

Materials & experimental systems

n/a	Involved in the study	
☒	☐	Antibodies
☒	☐	Eukaryotic cell lines
☒	☐	Palaeontology
☒	☐	Animals and other organisms
☒	☐	Human research participants
☒	☐	Clinical data

Methods

n/a	Involved in the study	
☒	☐	ChIP-seq
☒	☐	Flow cytometry
☒	☐	MRI-based neuroimaging
